# CALB2 Expression Is Associated with Tumor Progression and Prognosis in Colorectal Adenocarcinoma

**DOI:** 10.3390/genes17050510

**Published:** 2026-04-25

**Authors:** Feng Yang, Yizhou Liu, Na Luo, Qianxia Li, Guangyuan Hu

**Affiliations:** Department of Oncology, Tongji Hospital, Tongji Medical College, Huazhong University of Science and Technology, Wuhan 430030, China; d202382345@hust.edu.cn (F.Y.); m202576811@hust.edu.cn (Y.L.); luona0402@tjh.tjmu.edu.cn (N.L.)

**Keywords:** CALB2, colorectal adenocarcinoma, tumor progression, amino acid metabolism

## Abstract

**Background**: Globally, colorectal adenocarcinoma (COAD) continues to be one of the most frequent contributors to cancer-associated deaths, pointing out an ongoing demand for robust biomarkers capable of reflecting disease advancement and predicting patient outcomes. Calbindin 2 (CALB2), a calcium-binding protein, has been implicated in tumor progression in several malignancies, but the precise functional involvement of this molecule in COAD remains insufficiently characterized. This study aimed to examine the clinical relevance and functional significance of CALB2 in COAD. **Methods**: Clinical tissue-based analyses, bioinformatic analyses of publicly available datasets, in vitro and in vivo functional assays, and targeted metabolomic profiling were performed to evaluate CALB2 in COAD. **Results**: Immunofluorescence analysis of a COAD tissue microarray revealed that CALB2 expression was more frequently detected in stage III tumors and was associated with the proliferation marker Ki67. The Cancer Genome Atlas (TCGA) cohort’s bioinformatic analyses revealed an increase in CALB2 expression with advancing pathological T stage, and it was associated with poorer overall survival. Functional experiments demonstrated that CALB2 expression accelerated tumor growth in vivo and promoted the migration and proliferation of colorectal adenocarcinoma cells in vitro. At the molecular level, altered CALB2 expression was associated with changes in epithelial–mesenchymal transition-related markers, p53 Ser33 phosphorylation, and amino acid-related metabolic profiles. **Conclusions**: Collectively, these findings show a correlation between CALB2 expression and poor clinical outcomes and disease progression in colorectal adenocarcinoma.

## Simple Summary

Adenocarcinoma arising from the colorectum is a frequently diagnosed malignancy, and affected individuals with advanced-stage adenocarcinoma often experience an unfavorable prognosis. Identifying molecules that are linked to tumor progression may help improve prognosis assessment and guide future research. Calbindin 2 is a protein originally known for its role in calcium regulation, but its function in colorectal adenocarcinoma remains insufficiently characterized. This study examined Calbindin 2 expression in colorectal adenocarcinoma and investigated its biological relevance through the integration of clinical tissue samples, bioinformatic data, and both in vitro and in vivo experimental models. We found that Calbindin 2 expression was more frequently observed in advanced tumors and was associated with increased tumor cell growth and migration. Higher levels of Calbindin 2 were also linked to worse patient survival. In addition, Calbindin 2 expression was accompanied by changes in cellular behavior, p53 signaling activity, and amino acid metabolism. These findings suggest that Calbindin 2 may be useful for understanding tumor progression and could serve as a potential marker for disease severity in colorectal adenocarcinoma.

## 1. Introduction

With its high incidence rates and ranking as one of the primary causes of cancer-related deaths globally, colorectal adenocarcinoma (COAD) continues to pose a significant threat to global health [1,2,3]. Despite the clinical implementation of advanced surgical techniques and adjuvant regimens such as molecular targeted therapy and chemoradiotherapy, the long-term prognosis for patients—particularly those with metastatic disease—remains suboptimal [4,5]. According to the recent data, individuals with stage IV COAD continue to have a five-year survival rate of around 14%, underscoring the limited efficacy of current therapeutic strategies and garnering increased attention to the molecular mechanisms underlying treatment resistance [6]. Although early screening has contributed to a reduction in mortality, substantial limitations remain, indicating the need for the further investigation of genes associated with prognosis in colorectal adenocarcinoma [7,8].

Metabolic reprogramming represents a fundamental feature of malignant progression, whereby cancer cells restructure their metabolic circuitry to fuel accelerated growth and maintain viability despite the limited nutrient availability characteristic of the tumor milieu [9,10]. Among these metabolic alterations, a growing body of evidence has identified the amino acid metabolic pathways as pivotal drivers of neoplastic progression. Amino acids are essential substrates for protein biosynthesis and also serve as key precursors for the generation of glucosamine, nucleotides, polyamines, and glutathione [9]. Increasing evidence indicates that the dysregulation of amino acid metabolism sustains tumor growth and progression [11]. Studies have shown that aberrant amino acid utilization is a common feature of malignancies, including colon cancer, clear cell renal carcinoma, hepatocellular carcinoma, breast cancer, and ovarian cancer [12,13,14,15,16]. Cancer cells frequently adapt their amino acid acquisition and utilization strategies to sustain rapid proliferation and survival [17,18]. Emerging evidence indicates that COAD cells frequently exhibit aberrant amino acid profiles, such as the upregulation of asparagine, glutamate, and serine, which support biosynthetic demands and redox homeostasis [19,20,21]. Although dysregulated amino acid metabolism has been extensively reported in colorectal carcinoma, the molecular machinery responsible for these metabolic perturbations remains poorly defined.

Belonging to the family of calcium-binding proteins, Calbindin 2 (CALB2) has long been implicated in the maintenance of intracellular calcium balance and is frequently utilized as a histopathological marker across numerous disease contexts [22,23,24,25]. Recent studies have begun to hint at its involvement in tumor biology. Notably, evidence suggests that CALB2 facilitates the metastatic dissemination of pancreatic carcinoma through the inflammation-driven remodeling of the surrounding tumor stroma. In addition, CALB2 facilitates macrophage M2 polarization, thereby enhancing pancreatic cancer growth and metastatic potential [26]. Beyond its functional involvement in tumor progression, CALB2 also serves as a biomarker employed in diagnosing malignant pleural mesothelioma [27]. Interestingly, CALB2 expression is consistently absent in normal colonic epithelial cells, while its expression can be detected in colorectal cancer tissues [28]. Despite these observations, the biological function and mechanistic role of CALB2 in COAD cells remain largely unexplored.

In this study, we investigated the clinical relevance and functional role of CALB2 in colorectal adenocarcinoma using a combination of clinical tissue-based analyses, publicly available datasets with bioinformatic analyses, in vitro and in vivo functional assays, and targeted metabolomic profiling. By integrating these approaches, we aimed to characterize the association between CALB2 expression and tumor progression, explore its relationship with proliferative and migratory phenotypes, and examine potential links to amino acid metabolism and p53-related signaling pathways in colorectal adenocarcinoma.

## 2. Materials and Methods

### 2.1. Patient-Derived Tissue Samples

A tissue microarray (TMA) containing COAD specimens was purchased from SuperBioChips Co., Ltd., Shanghai, China. The microarray included tumor tissues from 30 patients diagnosed with COAD. According to the pTNM staging system (AJCC 7th edition), 19 cases were classified as stage III, while the remaining 11 cases were categorized as stage I–II. Each tissue core underwent fixation in formalin followed by paraffin embedding prior to subsequent analyses. The TMA was used for immunofluorescence analysis to assess the expression and spatial distribution of CALB2 and Ki67 in COAD tissues.

### 2.2. Multiplex Immunofluorescence Staining

Multiplex immunofluorescence was carried out based on a tyramide signal amplification (TSA)-based detection system. Deparaffinization of the formalin-fixed, paraffin-embedded (FFPE) slices was conducted using xylene, followed by a rehydration process involving a descending gradient of ethanol solutions. Antigen retrieval was then performed using a heat-mediated method, with tissue sections incubated in either an EDTA buffer (pH 9.0) or a citrate buffer (pH 6.0) for 25 min. To quench native catalytic activity, the slides were submerged in 3% hydrogen peroxide over a period of 10 min. To reduce background interference, the specimens were blocked using goat serum at ambient temperature for 20 min. For dual staining, the sections were treated with anti-CALB2 (1:500; Zen Bioscience, Shanghai, China; Cat# 200595) and anti-Ki67 (1:200; AIfang Biotech, Beijing, China; Cat# AF0019) rabbit polyclonal antibodies throughout the night at 4 °C.

After buffer rinses, HRP-linked polymer secondary antibodies were applied to the slides for 30 min at room temperature to facilitate detection. Visualization was achieved by reaction with TSA fluorophores (TYR-520 or TYR-620) for 5 min. For multiplex staining, the antibody–TSA complex was stripped via heat-mediated antigen retrieval, and the process was repeated for subsequent targets. Following a 10 min nuclear counterstaining process using DAPI, an anti-fade reagent was applied for sealing, and samples were then examined via fluorescence microscopy.

### 2.3. Survival Analysis

To examine the prognostic impact of CALB2, Kaplan–Meier plots were generated by utilizing genomic data from the TCGA-COAD database. The log-rank test was employed to compare survival outcomes between high-expression and low-expression clusters, which were established based on the median CALB2 quantity.

### 2.4. Gene Expression Differential Analysis

The profiles of gene expression were assessed by utilizing RNA-seq information retrieved from the TCGA repository. Expression matrices were generated after data preprocessing, and samples with incomplete clinical annotation were excluded from subsequent analyses. CALB2 expression levels were extracted for downstream comparisons.

Differential expression analyses were conducted between predefined sample groups. As the distribution of gene expression values deviated from normality, non-parametric statistical methods were applied. For comparisons between two independent groups, the Mann–Whitney U test was used. For comparisons among three or more groups, the Kruskal–Wallis test was employed. Only groups containing at least three samples were included in the analysis.

### 2.5. Cell Culture

For this study, HCT116, HT29, SW480, SW48, LOVO, and CACO2 (human-derived COAD lines) were sourced from the American Type Culture Collection (ATCC; Manassas, VA, USA). Cryopreserved cells (approximately 1 × 10^6^ cells per vial) were quickly thawed in a 37 °C water bath and then immediately transferred to 15 mL centrifuge tubes containing prewarmed complete culture medium. HCT116, HT29, SW480, and SW48 cells were grown in DMEM (Gibco, Waltham, MA, USA), while LOVO and CACO2 cells were cultured in RPMI-1640 medium (Gibco, Waltham, MA, USA). All media were supplemented with 10% fetal bovine serum (Gibco, Waltham, MA, USA) and 1% penicillin–streptomycin (HyClone, Logan, UT, USA) under standard culture conditions. Cells were collected by centrifugation at 1200 rpm for 5 min, resuspended, and seeded into T25 culture flasks (Corning, Corning, NY, USA). Cells were cultured under standard conditions at 37 °C in a humidified atmosphere containing 5% CO_2_.

Cells were routinely passaged using 0.25% trypsin–EDTA (Gibco, Waltham, MA, USA). HCT116 and HT29 cells were split at a ratio of 1:4 every 2–3 days, SW480 and SW48 at 1:3 every 3 days, and LOVO and CACO2 at 1:5 every 2 days. Cell counts were quantified with an automated cell counting system (Countstar, ALIT Life Science, Shanghai, China) according to the manufacturer’s instructions. Mycoplasma contamination was monitored monthly using the MycoAlert PLUS kit (Lonza, Basel, Switzerland). To ensure biological stability, assays utilized cells maintained for fewer than 20 passages, with medium replacement occurring every 48 h. Working cell stocks were generated at passages 3–5 using CryoStor CS10 freezing medium (StemCell Technologies, Vancouver, BC, Canada).

### 2.6. siRNA Transfection

Twenty-four hours prior to transfection, HCT116, HT29, SW480, and SW48 cells were plated in 24-well plates (Corning, Corning, NY, USA) at a seeding density of 1 × 10^5^ cells per well. Cells were cultured until approximately 70–90% confluence was reached. Lyophilized siRNAs targeting CALB2, along with a negative control siRNA (RiboBio, Guangzhou, China), were dissolved in RNase-free water to generate 20 μM stock solutions.

For transfection, 2 μL of InvitorRNA™ reagent (BIO-LOGY (Hubei) Biotechnology Co., Ltd., Wuhan, China) was first mixed with 50 μL of buffer, after which 2 μL of siRNA was added to the mixture. The specific siRNA oligonucleotide sequences are listed below: CALB2 siRNA-1 (st-h-CALB2_001, UGGGUAUAUUGAAGGUAAA), CALB2 siRNA-2 (st-h-CALB2_002, GUCAAAGAGUGACAACUUU), and a non-targeting control siRNA (UUCUCCGAACGUGUCACGUUU). After gentle mixing, the transfection mixture was maintained at 37 °C for 30 min to facilitate complex formation and was then introduced into the cells. Each well was brought to a final volume of 500 μL using complete culture medium, yielding a final siRNA concentration of 80 nM. Following transfection, specimens were collected after 1–3 days to evaluate silencing efficacy by qRT-PCR and Western blot. For pharmacological rescue experiments, cells were divided into three groups: NC, si1-CALB2, and si1-CALB2+PFT-α. The p53-specific inhibitor PFT-α hydrobromide (PFT-α, 10 μM; MCE, Monmouth Junction, NJ, USA) was introduced to the culture medium 24 h after siRNA transfection. Control groups (NC and si1-CALB2) were treated with an equivalent volume of DMSO as the vehicle control. All cells were then incubated for an additional 24 h before being harvested for RT-qPCR, Western blot, or functional assays (CCK-8 and wound-healing).

### 2.7. Plasmid Transfection

One day preceding the transfection process, cells were plated in 6-well plates (Corning, Corning, NY, USA) at 3 × 10^5^ cells per well in 2 mL of complete medium and allowed to grow to approximately 60–80% confluence. The culture medium was refreshed with complete medium 2 h prior to transfection. For complex preparation, 2 μg of plasmid DNA (the CALB2 overexpression plasmid constructed with the GV657 backbone, or the empty GV657 vector control; Wuhan Haimeng Biotechnology, Wuhan, China) was diluted in 100 μL of serum-free DMEM, followed by the addition of 2 μL of Neofect™ DNA transfection reagent (Neofect Biotech Co., Ltd., Beijing, China). Following a brief agitation, the transfection complexes were kept at 37 °C for a duration of 30 min, and then introduced into the culture system using a slow, dropwise approach. Cells were collected 24–48 h after transfection, and transfection efficacy was validated by qRT-PCR and Western blot.

### 2.8. Lentiviral Transfection

SW480 cells were infected with lentivirus carrying the CALB2 overexpression construct (generated using the pLVX-FLAG-P2A-Luc-T2A-Puro backbone) or the corresponding empty pLVX-FLAG-P2A-Luc-T2A-Puro control vector (Wuhan Haimeng Biotechnology, Wuhan, China) to generate stable cell lines. Cells were plated into 24-well culture plates at 3–5 × 10^4^ cells per well and maintained until they reached roughly 70% confluence. Lentiviral infection was carried out at an MOI of 20 in a culture medium supplemented with 5–10 μg/mL polybrene to facilitate viral entry. Cells were maintained overnight in the incubator.

At 24 h post-infection, the culture supernatant was removed and replaced with fresh complete medium. After 48 h, puromycin (1–10 μg/mL) was added to initiate antibiotic selection. Non-infected SW480 cells were used as controls to determine the appropriate puromycin concentration. The medium containing the selection antibiotic was renewed every 2–3 days until the control cells were fully eliminated, after which the stable CALB2-overexpressing clones were cultured and amplified for downstream analyses.

### 2.9. qRT-PCR

The HCT116, HT29, SW480, and SW48 cell lines were maintained in high-glucose DMEM containing 10% FBS at 37 °C with 5% CO_2_. When cultures reached 80–90% confluence, cells were replated into 6-well plates (3 × 10^5^ cells/well) and incubated for an additional 24 h before analysis. Total RNA was subsequently extracted using the TRIzol reagent (TaKaRa, Shiga, Japan).

The synthesis of cDNA was performed by employing HiScript II QRT SuperMix (Vazyme, Nanjing, China). Subsequently, qRT-PCR assays were executed utilizing 2× SYBR Green qPCR Master Mix II (Sevenbio, Beijing, China) according to the provided protocols. The specific primers used for amplification included the following: GAPDH (F: GACCACAGTCCATGCCATCA; R: GTCAAAGGTGGAGGAGTGGG), CALB2 (F: ACTTTGACGCAGACGGAAATG; R: GAAGTTCTCTTCGGTTGGCAG), p21 (CDKN1A) (F: TGTCCGTCAGAACCCATGC; R: AAAGTCGAAGTTCCATCGCTC), and BAX (F: CCCGAGAGGTCTTTTTCCGAG; R: CCAGCCCATGATGGTTCTGAT).

### 2.10. Western Blot Analysis

The HCT116, HT29, SW480, and SW48 cell lines were maintained in high-glucose DMEM containing 10% FBS at 37 °C with 5% CO_2_. When cell confluence reached approximately 80–90%, the cells were plated in 6-well culture plates (Corning, Corning, NY, USA) at a concentration of 3 × 10^5^ cells per well and maintained under standard conditions for 24 h before proceeding with protein isolation.

After washing the specimens twice with chilled PBS, cell lysis was performed on ice using RIPA buffer supplemented with 1% protease/phosphatase inhibitors (Servicebio, Wuhan, China). Cell lysates were incubated on ice for 30 min with gentle vortexing at 10 min intervals, followed by centrifugation at 12,000 rpm for 15 min at 4 °C. Following centrifugation, the supernatants were harvested as whole-cell protein extracts, and protein concentrations were determined using a BCA assay kit (Servicebio, Wuhan, China).

Uniform amounts of protein were separated via 10% SDS-PAGE before being electrotransferred onto PVDF membranes. After skim milk blocking, membranes were incubated at 4 °C overnight with primary antibodies against CALB2 (1:1000; Zen Bioscience, Shanghai, China; Cat# 200595), P53 (1:1000; Abclonal, Wuhan, China; Cat# A0263), phospho-P53 (Ser33) (1:1000; Abclonal, Wuhan, China; Cat# AP0762), E-cadherin (1:1000; Abclonal, Wuhan, China; Cat# A3044), N-cadherin (1:1000; Abclonal, Wuhan, China; Cat# A19083), β-catenin (1:1000; Abclonal, Wuhan, China; Cat# A19657), vimentin (1:1000; Abclonal, Wuhan, China; Cat# A19607), and GAPDH (1:10,000; Proteintech, Wuhan, China; Cat# 60004-1-Ig). After being treated with appropriate secondary antibodies for 1 h at ambient temperature, protein bands were detected using the West Pico Plus ECL reagent (Thermo Fisher Scientific, Waltham, MA, USA). For quantitative analysis, the density of the protein bands was determined using the software ImageJ (v1.53k, NIH, Bethesda, MD, USA). The expression levels of target proteins were normalized to GAPDH. For phosphorylation analysis, the relative levels of p-p53 were calculated as the ratio of p-p53 to total p53 intensity.

### 2.11. CCK8 Assay

Following detachment with 0.25% trypsin–EDTA (Gibco, Waltham, MA, USA), the HCT116, HT29, SW480, and SW48 cell lines were gathered and subjected to 5 min of centrifugation at 1200 rpm. Subsequently, an automated counter (Countstar; ALIT Life Science, Shanghai, China) was utilized to quantify cell populations. Specimens were redistributed into full growth media at a final concentration of 3 × 10^4^ cells/mL. Then, a 100 μL volume of this mixture (3 × 10^3^ cells) was added to the internal wells of Corning 96-well clusters (Corning, Corning, NY, USA), while the surrounding reservoirs were loaded with 100 μL PBS to mitigate fluid loss.

After cells were permitted to adhere for approximately 6–8 h, 10 μL of the CCK-8 solution (MCE, Monmouth Junction, NJ, USA) was carefully dispensed into each well. The plates were then maintained at 37 °C under light-protected conditions for 1–4 h. Absorbance was then recorded at 450 nm with 650 nm as the reference using a BioTek Synergy H1 microplate reader (BioTek, Winooski, VT, USA). Cellular viability was determined using the following formula: [(OD_450_ of treated samples − OD_450_ of blanks)/(OD_450_ of controls − OD_450_ of blanks)] × 100%.

### 2.12. Wound-Healing Assays

The HCT116, HT29, SW480, and SW48 cell lines were plated in 24-well culture plates and maintained under standard conditions for 24 h until a continuous cell monolayer was established. Seeding densities were adjusted for each cell line (HCT116, 2.5 × 10^5^ cells/well; HT29, 3 × 10^5^ cells/well; SW480, 2 × 10^5^ cells/well; SW48, 1.8 × 10^5^ cells/well). When cell confluence reached approximately 90–95%, scratches were made using a sterile 200 μL pipette tip held perpendicular to the plate. Cells were rinsed twice with PBS to clear debris, followed by the addition of serum-free medium.

Photographs were captured at 0, 24, and 48 h with a Leica DMi8 inverted microscope (Leica, Wetzlar, Germany) using a 10× objective lens. The same fields were photographed at each time point. Wound closure was measured using the software ImageJ (NIH, Bethesda, MD, USA) with the MRI Wound Healing Tool plugin and presented as the proportion of the healed area compared with the original wound size.

### 2.13. Transwell Migration Analysis

The HCT116, HT29, SW480, and SW48 cells were digested with 0.25% trypsin–EDTA and subsequently resuspended in DMEM containing 10% fetal bovine serum. Cell numbers and viability were checked using a Countstar IC1000 cell counter (ALIT Life Science, Shanghai, China), and only samples with viability above 95% were used. After rinsing with PBS, the specimens were redistributed into media devoid of serum to reach a final count of 5 × 10^5^ cells/mL.

For the migration assay, 8 μm pore-size inserts (Corning, Corning, NY, USA) were placed into 24-well plates. The basal reservoirs were filled with 500 μL DMEM containing 20% FBS, while 1 × 10^5^ cells in a 200 μL volume were inoculated into the apical inserts. The assembly was incubated at 37 °C with 5% CO_2_ for 24 h.

Post-incubation, top-layer cells were cleared using swabs. Migrated cells on the bottom were immobilized with 4% PFA for fifteen minutes and then dyed with 0.1% crystal violet for 20 min. Membranes were then washed with PBS to remove excess stain and air-dried at room temperature prior to analysis. Cell morphology and migration were examined using an inverted microscope (Leica DMi8, Leica Microsystems, Wetzlar, Germany) equipped with a 20× objective lens to capture representative images for analysis. Five fields were counted for each insert.

### 2.14. Amino Acid-Targeted Metabolomic Sequencing

Wild-type SW48 cells and CALB2-overexpressing SW48 cells were collected (1 × 10^7^ cells per sample). Frozen samples were placed on ice until thawed. To every specimen, 800 μL of a methanol–acetonitrile blend (1:1, *v*/*v*) enriched with isotopic internal standards was added. After vortexing for 30 s, the mixtures were sonicated in an ice–water bath for 5 min using a 30 s on/30 s off cycle. Samples were then stored at −20 degrees Celsius for 1 h and centrifuged at 14,000 revolutions per minute for 20 min at 4 degrees Celsius.

Following centrifugation, 700 μL of the supernatant was collected and evaporated to dryness using a vacuum concentrator. The residue was re-dissolved in 150 μL of acetonitrile/water (1:1, *v*/*v*) and centrifuged again to eliminate insoluble material. The resulting supernatant was subsequently subjected to LC–MS/MS analysis.

LC–MS/MS analysis was performed on a Q Exactive HF-X mass spectrometer (Thermo Fisher Scientific, Waltham, MA, USA) coupled to an ACQUITY UPLC HSS T3 column (Waters, Milford, MA, USA). The mobile phases were 0.1% formic acid in water (A) and 0.1% formic acid in acetonitrile (B). The gradient was 1% B for 1 min, 1% to 99% B from 1 to 12 min, 99% B from 12 to 14 min, and re-equilibration to 1% B until 17 min. Full-scan and MS/MS data were acquired at resolutions of 120,000 and 30,000, respectively. Three biological samples were analyzed, and each sample was injected twice.

### 2.15. Animal Care and Use

All procedures involving animals complied with the relevant institutional ethical standards and received formal approval from the Experimental Animal Welfare Ethics Committee of Tongji Hospital, Huazhong University of Science and Technology. Six-week-old nude mice were purchased from Jiangsu Jicui Experimental Animal Co., Ltd. (Nanjing, China) and housed under specific pathogen-free conditions in the animal facility of Tongji Hospital.

### 2.16. Subcutaneous Tumor Model

For the subcutaneous tumor model, six-week-old female nude mice were randomly assigned to the negative control (NC) or CALB2 overexpression (OE-CALB2) groups (n = 6 per group) using a simple randomization method based on a computer-generated random number sequence. This ensured that each animal had an equal probability of being assigned to either treatment group, thereby minimizing potential selection bias. Female nude mice (six weeks old) received a subcutaneous injection of 5 × 10^6^ cells, which was prepared in 100 μL PBS, into their dorsal axillary area. A total of six mice were assigned to every treatment group. Tumor formation was monitored after injection, and tumor size measurements were initiated on day 14. Tumor size was assessed every three to four days with calipers, and volume was calculated using the following formula: length × width^2^/2. At the study endpoint, mice were euthanized, and tumors were collected for further analysis.

### 2.17. Double Immunofluorescence Staining of Xenograft Tumors

To evaluate the spatial correlation between CALB2 expression and phosphorylated p53 in vivo, double immunofluorescence staining was performed on the excised subcutaneous tumor tissues. Tissues were fixed in 4% paraformaldehyde, embedded in paraffin, and sectioned at a 4 μm thickness. Following deparaffinization and rehydration, heat-mediated antigen retrieval was performed using citrate buffer (pH 6.0). The sections were blocked with 10% normal goat serum for 1 h. For dual staining, a tyramide signal amplification (TSA)-based sequential multiplexing method was employed to prevent cross-reactivity. Briefly, sections were first incubated with the primary antibody against CALB2 (1:500; Zen Bioscience, Shanghai, China; Cat# 200595), followed by visualization with a specific TSA fluorophore. After stripping the initial antibody complex via microwave heating, the sections were subsequently incubated with the primary antibody against p-p53-Ser33 (1:100; Abclonal, Wuhan, China; Cat# AP0762) and visualized with a distinct TSA fluorophore. Nuclei were counterstained with DAPI for 10 min. The slides were mounted with the anti-fade reagent, and images were captured using a fluorescence microscope (Leica DMi8; Leica Microsystems, Wetzlar, Germany).

### 2.18. Statistical Analysis

All statistical computations were performed using R (version 4.0.4, R Foundation for Statistical Computing, Vienna, Austria) and GraphPad Prism (version 8.0, GraphPad Software, San Diego, CA, USA). Prior to statistical testing, the normality of the data distribution for all continuous variables was assessed using the Shapiro–Wilk test. For in vitro cellular and molecular experiments, including Western blot densitometry, RT-qPCR, CCK-8, and migration assays, data are presented as mean ± SD from at least three independent biological replicates. The results of in vivo xenograft tumor growth curves and the analysis of clinical datasets (e.g., TCGA) are expressed as mean ± SEM.

For normally distributed variables, differences between two independent groups were evaluated using Student’s t-test, and differences among three or more groups (such as in the pharmacological rescue assays with PFT-α) were evaluated using one-way ANOVA followed by Tukey’s post hoc test. When the data did not meet the assumption of normal distribution, the non-parametric Wilcoxon rank-sum test (Mann–Whitney U test) or the Kruskal–Wallis test was employed.

All statistical tests were two-sided, and a *p* value of less than 0.05 was regarded as indicating statistical significance. Levels of significance were denoted as follows: * *p* < 0.05, ** *p* < 0.01, *** *p* < 0.001, and **** *p* < 0.0001.

## 3. Results

### 3.1. CALB2 Expression Is Associated with Tumor Progression in Colorectal Adenocarcinoma

**Figure 1:**
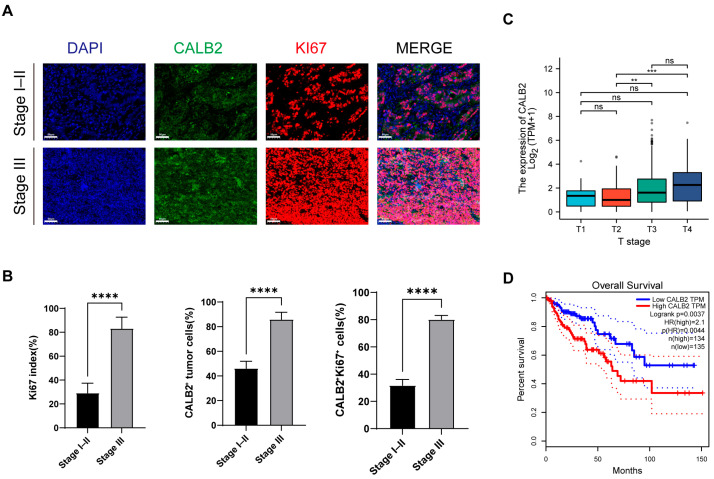
CALB2 expression is associated with tumor progression in colorectal adenocarcinoma. (**A**) Representative immunofluorescence images of CALB2 (green) and Ki67 (red) in colorectal adenocarcinoma tissues from stage I–II (n = 11) and stage III (n = 19) patients. Nuclei were stained with DAPI (blue). Scale bar = 50 μm. (**B**) Quantification of Ki67 index, CALB2-positive tumor cells, and CALB2^+^Ki67^+^ double-positive cells in colorectal adenocarcinoma tissues. (**C**) CALB2 mRNA expression across pathological T stages in colorectal adenocarcinoma based on data from The Cancer Genome Atlas (TCGA) COAD cohort. Grey dots represent individual outlier samples beyond the whiskers, and darker or black dots indicate overlapping individual data points rather than a separate group. (**D**) Kaplan–Meier analysis of overall survival in patients with colorectal adenocarcinoma from the TCGA COAD cohort stratified by CALB2 expression level (** *p* < 0.01, *** *p* < 0.001, **** *p* < 0.0001).

The expression of CALB2 in colorectal adenocarcinoma was examined by immunofluorescence staining using a tissue microarray (TMA) comprising 30 tumor specimens, including 11 stage I–II cases and 19 stage III cases. Immunofluorescence analysis showed heterogeneous CALB2 staining patterns within tumor tissues. Numerous CALB2^+^Ki67^+^ double-positive cells were observed in tumor regions, indicating the co-expression of CALB2 and the proliferation marker Ki67 (Figure 1A).

When tumors were stratified according to clinical stage, stage III specimens exhibited a higher abundance of Ki67-positive cells when compared with stage I–II specimens. Correspondingly, a greater number of CALB2/Ki67 double-positive cells was observed in stage III tumors (Figure 1A). Quantitative analysis demonstrated that the Ki67 index was significantly higher in stage III tumors than in stage I–II tumors (Figure 1B, left panel). In addition, the proportion of CALB2-positive tumor cells was higher in stage III tumors when compared with stage I–II tumors (Figure 1B, middle panel). The percentage of CALB2^+^Ki67^+^ double-positive cells was also increased in stage III tumors relative to stage I–II tumors (Figure 1B, right panel). Furthermore, we investigated the correlation between CALB2 expression and key clinicopathological characteristics in this cohort. As summarized in Supplementary Table S1, CALB2 expression levels showed no significant association with patient age (*p* = 0.482), gender (*p* = 0.865), or tumor size (*p* = 0.526). These data indicate that, within the analyzed samples, CALB2 upregulation was not notably biased by baseline patient demographics or primary tumor dimensions.

To further characterize CALB2 expression, transcriptomic data from The Cancer Genome Atlas (TCGA) colorectal adenocarcinoma (COAD) cohort were analyzed. CALB2 mRNA expression increased across pathological T stages, with higher expression levels observed in higher T-stage tumors (Figure 1C). Survival analysis using the Kaplan–Meier method in the TCGA colorectal adenocarcinoma cohort demonstrated that individuals with elevated CALB2 expression exhibited reduced overall survival relative to those with lower expression levels (Figure 1D).

### 3.2. CALB2 Promotes Proliferation of Colorectal Adenocarcinoma Cells In Vitro

**Figure 2:**
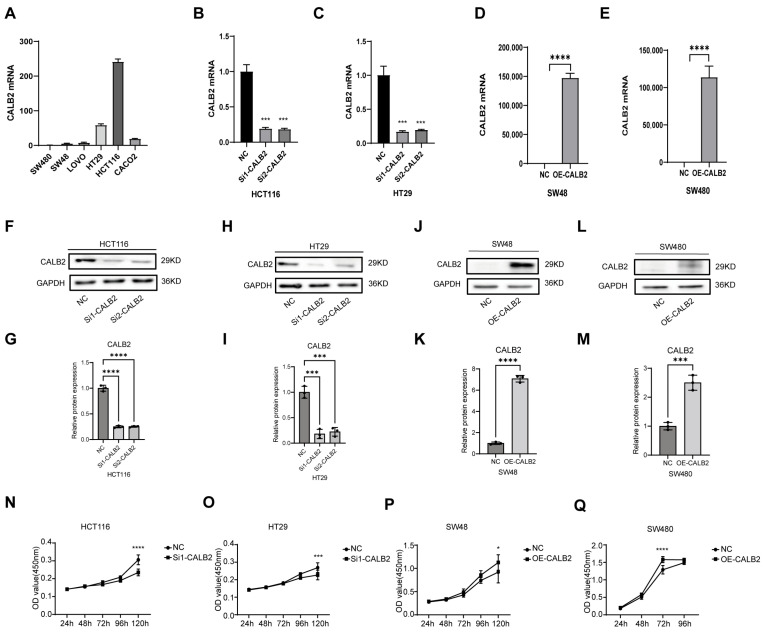
CALB2 promotes proliferation of colorectal adenocarcinoma cells in vitro. (**A**) Relative CALB2 mRNA expression levels in a panel of colorectal adenocarcinoma cell lines (SW480, SW48, LOVO, HT29, HCT116, and CACO2), as determined by qRT-PCR. (**B**,**C**) CALB2 mRNA expression in HCT116 (**B**) and HT29 (**C**) cells at 24 h following transfection with two independent CALB2 siRNAs (si1-CALB2 and si2-CALB2) or negative control (NC). (**D**,**E**) CALB2 mRNA expression in SW48 (**D**) and SW480 (**E**) cells after CALB2 overexpression (OE-CALB2) or negative control (NC). (**F**,**H**) Western blot analysis of CALB2 protein levels in HCT116 (**F**) and HT29 (**H**) cells after CALB2 knockdown. (**G**,**I**) Densitometric quantification of the Western blot results shown in panels (**F**) and (**H**), respectively. (**J**,**L**) Western blot analysis of CALB2 protein levels in SW48 (**J**) and SW480 (**L**) cells following CALB2 overexpression. (**K**,**M**) Densitometric quantification of the Western blot results shown in panels J and L, respectively. (**N**,**O**) Cell proliferation of HCT116 (**N**) and HT29 (**O**) cells after CALB2 knockdown(using si1-CALB2), assessed by CCK-8 assays at the indicated time points. (**P**,**Q**) Cell proliferation of SW48 (**P**) and SW480 (**Q**) cells following CALB2 overexpression, assessed by CCK-8 assays at the indicated time points. (n = 3, * *p* < 0.05, *** *p* < 0.001, **** *p* < 0.0001).

CALB2 expression was first examined in a panel of colorectal adenocarcinoma cell lines, including HCT116, HT29, SW480, SW48, LOVO, and CACO2. Higher endogenous CALB2 expression was detected in HCT116 and HT29 cells, whereas lower expression levels were observed in SW480 and SW48 cells (Figure 2A). Based on these expression patterns, HCT116 and HT29 cells were selected for CALB2 knockdown, while SW480 and SW48 cells were used for CALB2 overexpression in subsequent experiments.

CALB2 expression was efficiently reduced in HCT116 and HT29 cells using two independent siRNAs at 24 h post-transfection, whereas CALB2 was successfully overexpressed in SW480 and SW48 cells by plasmid transfection. Alterations in CALB2 expression were confirmed at both the mRNA and protein levels and further validated using densitometric quantification (Figure 2B–M). Furthermore, a time-course qRT-PCR analysis evaluating CALB2 knockdown efficiency in HCT116 cells revealed that maximal mRNA suppression was achieved at 24 h post-transfection, and this significant knockdown effect was stably maintained between 48 and 72 h (Supplementary Figure S1).

Cell proliferation was assessed using CCK-8 assays. CALB2 knockdown (using si1-CALB2) significantly suppressed the proliferative capacity of HCT116 and HT29 cells (Figure 2N–O). Importantly, functional assays utilizing the second independent siRNA (si2-CALB2) produced consistent inhibitory effects on cell proliferation, effectively ruling out potential off-target effects (Supplementary Figure S2). In contrast, CALB2 overexpression markedly enhanced cell proliferation in SW480 and SW48 cells (Figure 2P–Q). Together, these findings indicate that CALB2 positively regulates colorectal adenocarcinoma cell growth in vitro.

### 3.3. CALB2 Enhances Migration of Colorectal Adenocarcinoma Cells In Vitro

**Figure 3:**
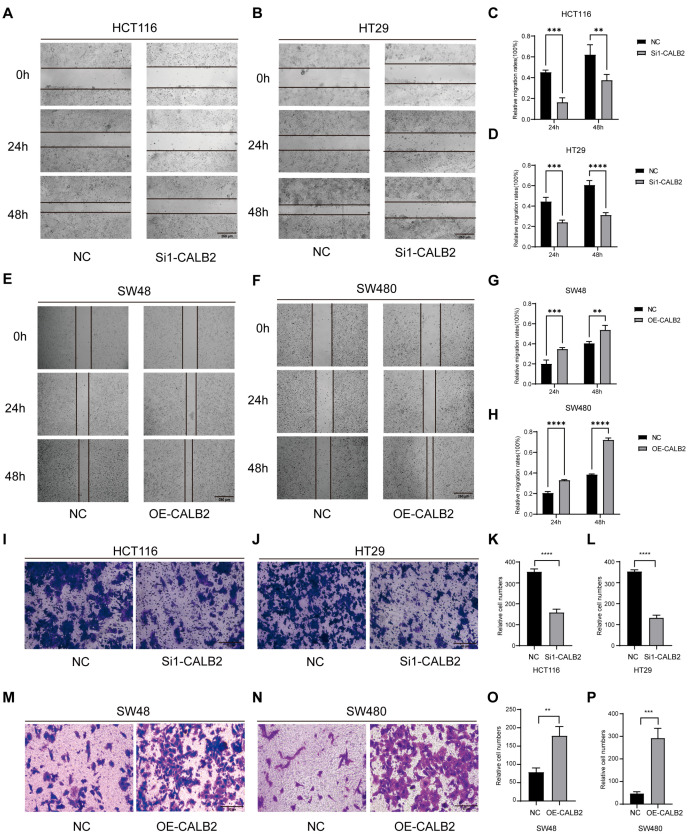
CALB2 enhances migration of colorectal adenocarcinoma cells in vitro. (**A**,**B**) Wound-healing assays performed in HCT116 (**A**) and HT29 (**B**) cells transfected with negative control (NC) or CALB2 siRNA (si1-CALB2). Representative images were captured at 0, 24, and 48 h after scratching. (**C**,**D**) Quantification of wound closure in HCT116 (**C**) and HT29 (**D**) cells at 24 and 48 h following CALB2 knockdown (using si1-CALB2). (**E**,**F**) Wound-healing assays in SW48 (**E**) and SW480 (**F**) cells transfected with NC or CALB2 overexpression plasmid (OE-CALB2). Images were obtained at 0, 24, and 48 h after scratching. (**G**,**H**) Quantification of wound closure in SW48 (**G**) and SW480 (**H**) cells at 24 and 48 h following CALB2 overexpression. (**I**,**J**) Transwell migration assays in HCT116 (**I**) and HT29 (**J**) cells after CALB2 knockdown (using si1-CALB2). (**K**,**L**) Quantification of migrated cells in HCT116 (**K**) and HT29 (**L**) cells following CALB2 knockdown (using si1-CALB2). (**M**,**N**) Transwell migration assays in SW48 (**M**) and SW480 (**N**) cells after CALB2 overexpression. (**O**,**P**) Quantification of migrated cells in SW48 (**O**) and SW480 (**P**) cells following CALB2 overexpression (n = 3, ** *p* < 0.01, *** *p* < 0.001, **** *p* < 0.0001).

The effect of CALB2 on cell migration was first assessed using wound-healing assays. Silencing of CALB2 (using si1-CALB2) markedly delayed scratch closure in HCT116 and HT29 cells, indicating reduced migratory ability. In contrast, accelerated wound closure was observed in CALB2-overexpressing SW480 and SW48 cells (Figure 3A–H).

The migratory effect of CALB2 was further evaluated using Transwell migration assays. CALB2 knockdown (using si1-CALB2) in HCT116 and HT29 cells resulted in a significant reduction in the number of migrated cells (Figure 3I–L). Importantly, these anti-migratory effects were consistently observed using the second independent siRNA (si2-CALB2) in both wound-healing and Transwell assays, further validating the specificity of CALB2 in regulating cell migration (Supplementary Figure S3). Conversely, enforced CALB2 expression in SW480 and SW48 cells significantly increased their migration ability (Figure 3M–P).

Together, these results demonstrate that CALB2 enhances the migratory capacity of colorectal adenocarcinoma cells in vitro.

### 3.4. CALB2 Overexpression Promotes Tumor Growth In Vivo

**Figure 4:**
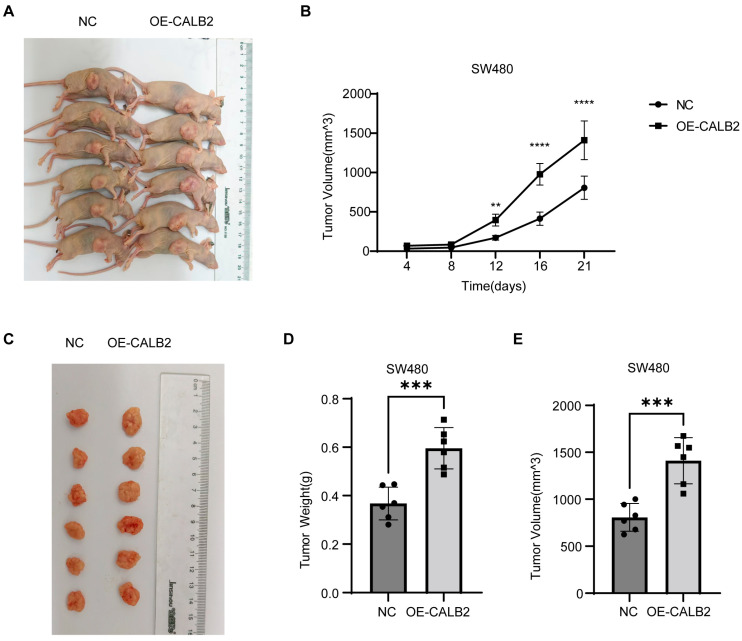
CALB2 overexpression promotes tumor growth in vivo. (**A**) Nude mice bearing subcutaneous tumors derived from SW480 cells transfected with negative control (NC) or CALB2 overexpression vector (OE-CALB2). (**B**) Tumor growth curves showing tumor volume changes over time in mice injected with NC or OE-CALB2 SW480 cells. (**C**) Excised tumors from the NC and OE-CALB2 groups at the experimental endpoint. (**D**) Comparison of tumor weights between NC and OE-CALB2 groups. (**E**) Comparison of final tumor volumes between NC and OE-CALB2 groups (n = 6, ** *p* < 0.01, *** *p* < 0.001, **** *p* < 0.0001).

To evaluate the effect of CALB2 on tumor growth in vivo, SW480 cells stably overexpressing CALB2 (OE-CALB2) or negative control (NC) cells were subcutaneously injected into nude mice. Representative images showed that tumors derived from OE-CALB2 cells were visibly larger than those from NC cells at the experimental endpoint (Figure 4A,C).

Tumor growth was monitored over time, and tumor volumes were measured at the indicated time points. Tumors in the OE-CALB2 group exhibited a significantly faster growth rate compared with the NC group, with a marked increase in tumor volume observed from day 12 onward (Figure 4B).

At the experimental endpoint, tumors were excised and quantitatively analyzed. Tumors derived from OE-CALB2 cells showed a significantly higher tumor weight than those from the NC group (Figure 4D). Consistently, tumors in the OE-CALB2 group exhibited a significantly greater final volume than those in the control group at the experimental endpoint (Figure 4E).

### 3.5. Association of CALB2 Expression with EMT-Related Molecular Markers in Colorectal Adenocarcinoma

**Figure 5:**
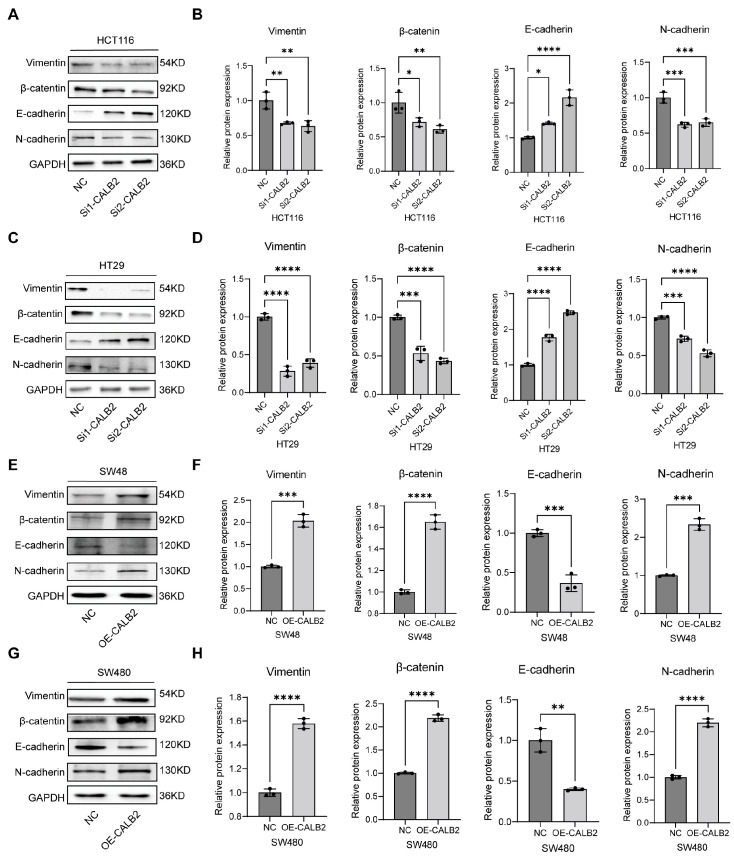
Association of CALB2 Expression with EMT-Related Molecular Markers in Colorectal Adenocarcinoma. (**A**,**B**) Representative Western blot images (**A**) and densitometric quantification (**B**) of epithelial (E-cadherin) and mesenchymal markers (vimentin, β-catenin, and N-cadherin) in HCT116 cells following CALB2 knockdown using two independent siRNAs (si1-CALB2 and si2-CALB2) or negative control (NC). (**C**,**D**) Representative Western blot images (**C**) and densitometric quantification (**D**) of EMT-related proteins in HT29 cells after CALB2 knockdown using two independent siRNAs or negative control (NC). (**E**,**F**) Representative Western blot images (**E**) and densitometric quantification (**F**) of EMT-related proteins in SW48 cells following CALB2 overexpression (OE-CALB2) or negative control (NC). (**G**,**H**) Representative Western blot images (**G**) and densitometric quantification (**H**) of EMT-related proteins in SW480 cells after CALB2 overexpression (OE-CALB2) or negative control (NC). GAPDH was used as an internal loading control. Bar graphs represent the relative protein expression levels normalized to GAPDH. (n = 3, * *p* < 0.05, ** *p* < 0.01, *** *p* < 0.001, **** *p* < 0.0001).

To investigate the impact of CALB2 on the epithelial–mesenchymal transition (EMT) process, Western blot analysis and subsequent densitometric quantification were performed in multiple colorectal adenocarcinoma cell lines. In HCT116 and HT29 cells, CALB2 silencing (via si1-CALB2 and si2-CALB2) led to a significant upregulation of the epithelial marker E-cadherin, while the expression levels of mesenchymal markers, including vimentin, β-catenin, and N-cadherin, were markedly reduced. The statistical analysis of the protein-to-GAPDH ratios confirmed that these alterations were highly significant, demonstrating that CALB2 knockdown effectively suppresses the mesenchymal phenotype (Figure 5A–D).

Conversely, the stable overexpression of CALB2 in SW48 and SW480 cells significantly promoted the EMT program. As shown by the immunoblotting results and corresponding quantitative data, CALB2 overexpression resulted in a substantial decrease in E-cadherin expression, accompanied by a robust increase in vimentin, β-catenin, and N-cadherin levels (Figure 5E–H). Collectively, these findings demonstrate that CALB2 expression is positively correlated with the mesenchymal state and negatively correlated with the epithelial state, suggesting that CALB2 is a key molecular driver of EMT in colorectal adenocarcinoma cells.

### 3.6. Association of CALB2 Expression with p53 Signaling in Colorectal Adenocarcinoma

**Figure 6:**
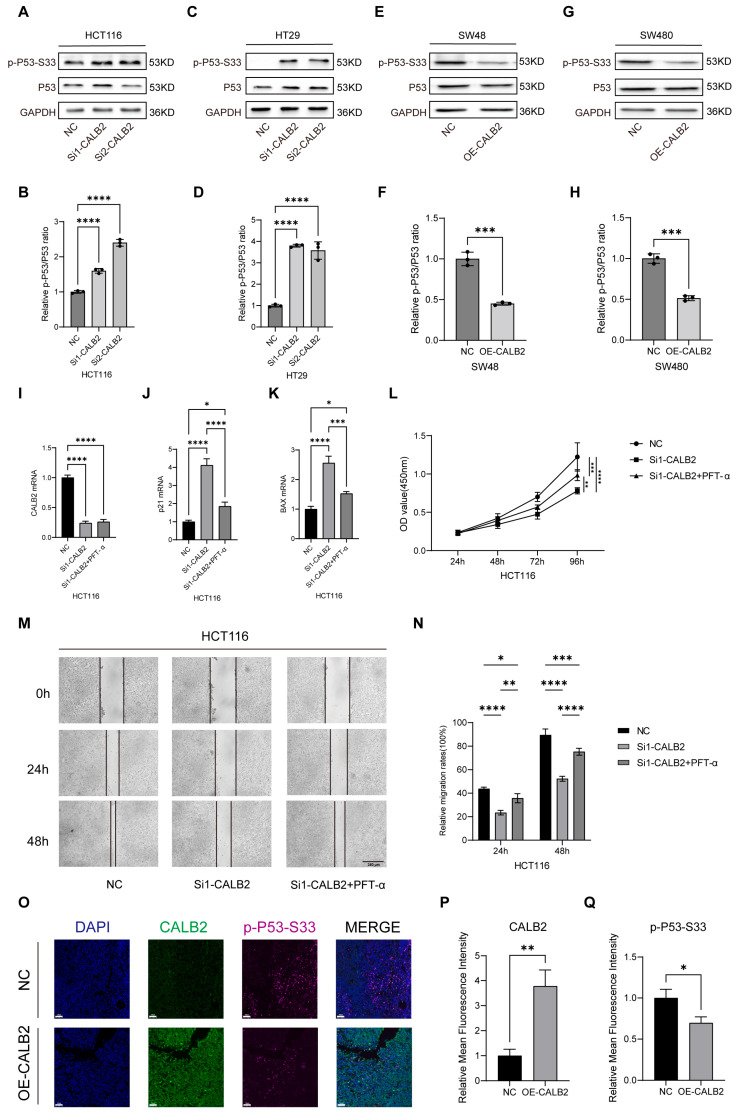
Association of CALB2 expression with p53 signaling in colorectal adenocarcinoma. (**A**,**B**) Western blot analysis of p-p53-Ser33 and total p53 protein levels (**A**) and corresponding densitometric quantification (**B**) in HCT116 cells following CALB2 knockdown using two independent siRNAs (si1-CALB2 and si2-CALB2) or negative control (NC). (**C**,**D**) Western blot analysis of p-p53-Ser33 and total p53 protein levels (**C**) and corresponding densitometric quantification (**D**) in HT29 cells after CALB2 knockdown or negative control (NC). (**E**,**F**) Western blot analysis of p-p53-Ser33 and total p53 protein levels (**E**) and corresponding densitometric quantification (**F**) in SW48 cells after CALB2 overexpression (OE-CALB2) or negative control (NC). (**G**,**H**) Western blot analysis of p-p53-Ser33 and total p53 protein levels (**G**) and corresponding densitometric quantification (**H**) in SW480 cells after CALB2 overexpression (OE-CALB2) or negative control (NC). (**I**–**K**) RT-qPCR analysis of CALB2 (**I**), p21(CDKN1A) (**J**), and BAX (**K**) mRNA levels in HCT116 cells transfected with NC, si1-CALB2, or si1-CALB2 combined with the p53 inhibitor PFT-α (10 μM). (**L**) CCK-8 assay of HCT116 cells in the NC, si1-CALB2, and si1-CALB2+PFT-α groups over a 96 h period. (**M**,**N**) Representative images of scratch wound-healing assays (**M**) and quantitative analysis of relative migration rates (**N**) in HCT116 cells at 0, 24, and 48 h post-scratching. Scale bar = 250 μm. (**O**–**Q**) Representative double immunofluorescence staining (**O**) and quantitative analysis of fluorescence intensity (**P**,**Q**) for CALB2 and p-p53-Ser33 in xenograft tumor tissues from NC and OE-CALB2 groups. Scale bar = 50 μm. (n = 3, * *p* < 0.05, ** *p* < 0.01, *** *p* < 0.001, **** *p* < 0.0001).

Western blot analysis revealed significant alterations in p53 signaling following the modulation of CALB2 expression in colorectal adenocarcinoma cells. In HCT116 and HT29 cells, CALB2 silencing led to a marked increase in p53 phosphorylation at the Ser33 residue, while total p53 protein levels remained largely unchanged (Figure 6A,C). Densitometric quantification further confirmed that the relative p-p53(Ser33)/p53 ratio was significantly elevated upon CALB2 knockdown (*p* < 0.0001; Figure 6B,D). In contrast, CALB2 overexpression in SW480 and SW48 cells significantly reduced p53 Ser33 phosphorylation, as evidenced by a substantial decrease in the p-p53/p53 ratio (Figure 6E–H).

To further determine whether the oncogenic effects of CALB2 are functionally dependent on the p53 pathway, we performed a pharmacological rescue assay using the p53-specific inhibitor Pifithrin-α (PFT-α) in HCT116 cells. First, the efficacy of the inhibitor was validated via RT-qPCR. While CALB2 knockdown (using si1-CALB2) induced a significant upregulation of the canonical p53 targets p21 (CDKN1A) and BAX, this induction was effectively attenuated following PFT-α treatment (Figure 6I–K), confirming the successful suppression of p53 transcriptional activity.

Subsequently, we evaluated the biological impact of this regulatory axis. CCK-8 assays demonstrated that the inhibition of cell proliferation induced by CALB2 knockdown was significantly reversed by PFT-α treatment (Figure 6L). Similarly, wound-healing assays revealed that the reduced migratory capacity of CALB2-deficient cells was partially restored upon p53 inhibition (Figure 6M,N). Collectively, these findings provide robust functional evidence that CALB2-driven malignant progression is mediated, at least in part, through the antagonism of the p53 signaling pathway.

To further validate these in vitro mechanistic findings within the in vivo tumor microenvironment, we performed double immunofluorescence staining for both CALB2 and p-p53-Ser33 on the excised xenograft tumor tissues. Consistent with the Western blot results, tumors derived from OE-CALB2 cells exhibited robust CALB2 expression accompanied by reduced p-p53-Ser33 fluorescence intensity compared with the NC group, which showed low CALB2 but prominent p-p53-Ser33 staining (Figure 6O–Q). This in vivo spatial evidence further corroborates the notion that elevated CALB2 suppresses the activation of p53 signaling during tumor progression.

### 3.7. CALB2 Overexpression Is Associated with Altered Amino Acid Metabolism in Colorectal Adenocarcinoma

**Figure 7:**
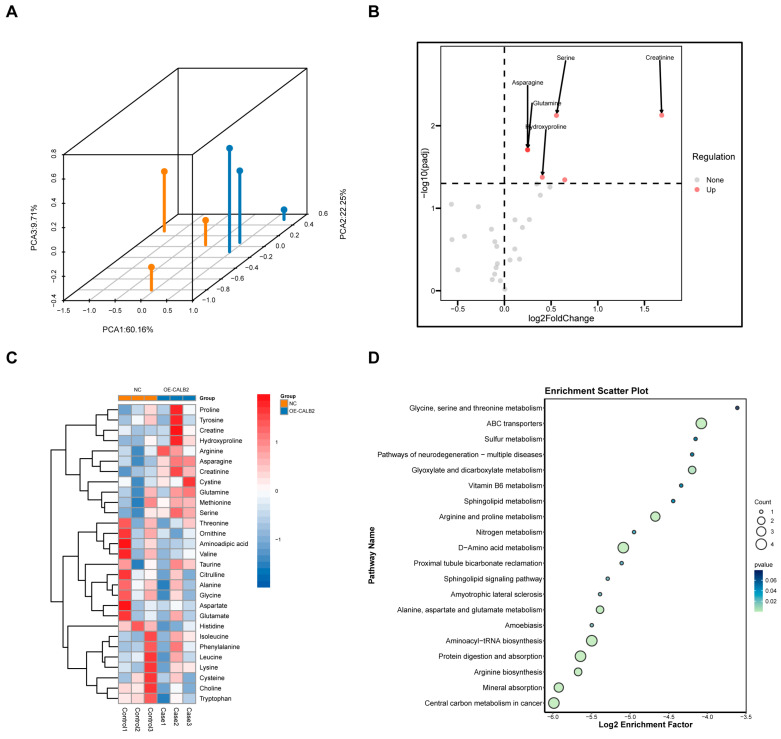
CALB2 overexpression is associated with altered amino acid metabolism in colorectal adenocarcinoma. (**A**) Three-dimensional PCA plot showing the separation between control (NC) and CALB2-overexpressing (OE-CALB2) SW48 cells. Orange and blue dots represent samples from the NC and OE-CALB2 groups, respectively. Each dot represents one biological replicate. (**B**) Volcano plot of differentially regulated metabolites between NC and OE-CALB2 SW48 cells. (**C**) Heatmap of differential metabolites in NC and OE-CALB2 SW48 cells. (**D**) Pathway enrichment analysis of differential metabolites in SW48 cells (n = 3).

To investigate the impact of CALB2 on amino acid metabolism in colorectal adenocarcinoma cells, targeted amino acid metabolomic profiling was performed in SW48 cells following CALB2 overexpression. A total of 31 amino acid-related metabolites were detected. Principal component analysis (PCA) revealed a clear separation between the CALB2 overexpression and control groups, indicating distinct global amino acid metabolic profiles (Figure 7A).

Among the detected metabolites, five metabolites showed significantly higher abundance following CALB2 overexpression: asparagine, creatinine, hydroxyproline, serine, and glutamine (Figure 7B,C). These alterations indicate a selective shift in amino acid-related metabolic profiles associated with increased CALB2 expression.

Pathway enrichment analysis based on the Kyoto Encyclopedia of Genes and Genomes (KEGG) database revealed that metabolites altered by CALB2 overexpression were mainly enriched in pathways related to amino acid biosynthesis, ABC transporter activity, and aminoacyl-tRNA biosynthesis (Figure 7D). Together, these results demonstrate that elevated CALB2 expression is accompanied by significant changes in the amino acid metabolic pathways in colorectal adenocarcinoma cells.

## 4. Discussion

Colorectal adenocarcinoma (COAD) remains a formidable challenge in clinical oncology. While progress in diagnostic and screening technologies has led to better early identification and therapeutic success in selected patient groups, a significant proportion of patients with COAD are still diagnosed at locally advanced or metastatic stages [29,30]. Furthermore, the clinical response of advanced-stage disease to systemic therapies exhibits substantial heterogeneity, often complicated by the emergence of primary or acquired resistance, which limits long-term survival benefits [31]. Despite the implementation of multimodal treatment approaches, such as surgical resection, cytotoxic chemotherapy, and targeted therapeutic agents, survival outcomes have improved only in selected patient subsets, and the long-term overall outlook for patients diagnosed with metastatic colorectal adenocarcinoma continues to be poor [32]. Thus, the discovery of previously unrecognized biomolecular indicators is urgently required to more accurately predict prognosis, optimize risk stratification, and provide a biological foundation for the development of innovative therapeutic targets.

Calbindin 2 (CALB2), a prominent member of the EF-hand domain family, is traditionally recognized for its physiological roles in intracellular calcium buffering and signal transduction [33]. However, emerging evidence suggests that CALB2 is more than a passive “calcium sensor”; its aberrant upregulation in various epithelial malignancies indicates a functional evolution into a critical survival factor and a regulator of tumor aggressiveness [34,35,36]. For instance, recent studies have identified CALB2 as a key initiator of the TRPV2-Ca2+-ERK1/2 signaling cascade, which actively drives the metastatic process in hepatocellular carcinoma [34]. Consistent with this, our exploratory analysis of 30 COAD specimens showed a trend where CALB2 protein expression was more prevalent in stage III tissues and frequently overlapped with the proliferation marker Ki67, suggesting a potential link between CALB2 and tumor cell proliferation. Notably, within this cohort, CALB2 expression appeared to be independent of clinicopathological parameters such as age and tumor size. To further evaluate the clinical significance of these findings, we utilized the large-scale TCGA-COAD cohort, which provided more robust evidence that CALB2 mRNA levels significantly correlate with advancing the pathological T stage and reduced overall survival. Collectively, these observations suggest that elevated CALB2 levels are associated with malignant progression and unfavorable outcomes in colorectal adenocarcinoma.

In line with its clinical relevance, our in vitro functional assays demonstrated that CALB2 is involved in the regulation of malignant behaviors in colorectal adenocarcinoma (COAD) cells. CALB2 knockdown significantly reduced cell proliferation and migratory capacity in HCT116 and HT29 cells, whereas CALB2 overexpression in SW480 and SW48 cells resulted in enhanced proliferative and migratory abilities. These in vitro findings were further supported by in vivo xenograft experiments, in which CALB2 overexpression markedly accelerated tumor growth, as reflected by the increased tumor volume and tumor weight in nude mice. Together, these results indicate that CALB2 contributes to tumor growth and progression in colorectal adenocarcinoma. At the molecular level, alterations in CALB2 expression were accompanied by coordinated changes in epithelial–mesenchymal transition (EMT)-related markers. In particular, an alteration in CALB2 levels was linked to the downregulation of the epithelial marker E-cadherin, along with the increased expression of proteins linked to the mesenchymal phenotype, including N-cadherin, β-catenin, and vimentin. Notably, these molecular shifts provide a compelling rationale for the accelerated wound closure and enhanced collective migration captured in our functional assays. EMT is broadly acknowledged as a fundamental mechanism driving cancer invasion and metastatic dissemination and therapeutic resistance in colorectal cancer [37,38,39]. The observed EMT-related molecular changes provide a potential explanation for the enhanced migratory behavior and tumor growth associated with elevated CALB2 expression.

Given the pronounced effects of CALB2 on proliferation, migratory capacity, and EMT-related molecular features in colorectal adenocarcinoma cells, it was reasonable to further examine whether CALB2 is associated with the key tumor suppressor signaling pathways. The p53 pathway is fundamentally involved in governing cellular proliferation, migration, and EMT in multiple solid tumors, and its functional dysregulation is closely linked to tumor progression [40]. Approximately 43% of colorectal carcinomas carry mutations in the TP53 gene, highlighting the critical role of this signaling pathway in tumorigenesis [41]. Previous studies have shown that the phosphorylation of p53 at the Ser33 residue is associated with its pro-apoptotic activity [42]. In this study, changes in CALB2 expression were closely associated with alterations in p53 Ser33 phosphorylation, with CALB2 silencing enhancing Ser33 phosphorylation and CALB2 overexpression showing the opposite trend. One possible mechanism is that CALB2 sequesters intracellular calcium ions, thereby inhibiting the activation of calcium-dependent kinases (e.g., CaMKII, p38 MAPK, and JNK) required for p53 Ser33 phosphorylation [43,44,45,46]. To further explore this functional link, we performed pharmacological rescue experiments using the p53-specific inhibitor PFT-α. Our results showed that the suppression of p53 transcriptional activity partially attenuated the tumor-suppressive effects induced by CALB2 knockdown. These functional findings, combined with the inverse spatial correlation observed in our xenograft immunofluorescence analysis, suggest that the CALB2/p53 axis may be a relevant regulatory mechanism in COAD progression. By potentially suppressing p53 activation, elevated CALB2 levels might facilitate the evasion of apoptosis and cell cycle arrest, thereby contributing to the malignant phenotype of colorectal adenocarcinoma. While the precise upstream kinase cascade requires further definitive characterization, our data point toward CALB2 as a potential negative regulator of p53 signaling and suggest its value as a candidate therapeutic target.

Metabolic reprogramming constitutes a defining characteristic of tumor advancement, empowering cancer cells to satisfy elevated anabolic and energy requirements within hostile microenvironments [47,48]. Among these alterations, the metabolism of amino acids has become increasingly recognized as a key contributor to tumor growth, redox homeostasis, and survival [49]. In this study, targeted metabolomic analysis revealed that CALB2 overexpression was associated with distinct changes in amino acid-related metabolic profiles in colorectal adenocarcinoma cells.

Specifically, CALB2 overexpression led to increased levels of several amino acid-related metabolites, including asparagine, serine, glutamine, hydroxyproline, and creatinine. These metabolites are known to support multiple aspects of tumor biology. Glutamine and serine play central roles in nucleotide biosynthesis, redox balance, and anabolic metabolism, while asparagine has been documented to support cancer cell viability under conditions of metabolic stress [50,51]. The coordinated elevation of these metabolites suggests that CALB2 expression is associated with a metabolic state favorable for sustained proliferation and cellular adaptation.

Pathway enrichment analysis further indicated that CALB2-associated metabolic alterations were mainly enriched in amino acid biosynthesis, ABC transporter pathways, and aminoacyl-tRNA biosynthesis. These pathways are closely linked to enhanced nutrient uptake, protein synthesis, and translational capacity, which are essential for rapidly proliferating cancer cells [52,53,54]. Together, these findings suggest that CALB2 overexpression may be accompanied by a broader remodeling of amino acid utilization and transport rather than isolated changes in individual metabolites.

A well-established function of p53 is its involvement in regulating cellular metabolism, which encompasses the preservation of amino acid homeostasis and the integration of stress-responsive signaling pathways [55]. In this context, the association between CALB2 expression, altered p53 signaling activity, and amino acid metabolic changes observed in our study raises the possibility that these processes may be functionally interconnected. However, whether CALB2 directly regulates metabolic enzymes or influences amino acid metabolism through p53-dependent or p53-independent mechanisms requires further investigation.

Importantly, while the present data support a close association between CALB2 expression and aggressive tumor behavior, the precise molecular mechanisms underlying these observations remain to be fully elucidated. Additionally, although our in vivo experiments successfully utilized a gain-of-function model to demonstrate the oncogenic potential of CALB2, the impact of stable CALB2 depletion on tumor progression in a physiological microenvironment was not evaluated. Therefore, future investigations employing shRNA-mediated stable silencing in vivo are essential to fully validate its loss-of-function effects. Furthermore, considering the high genetic heterogeneity of colorectal adenocarcinoma, it remains to be determined how the oncogenic role of CALB2 is modulated by specific genetic backgrounds, such as the mutational status of TP53. Further studies are warranted to determine whether CALB2 directly regulates key signaling and metabolic pathways or functions as a component of broader oncogenic programs in colorectal adenocarcinoma. Nonetheless, our findings suggest that CALB2 may represent a clinically relevant biomarker of disease progression and a potential entry point for mechanistic and therapeutic exploration in colorectal adenocarcinoma.

## 5. Conclusions

This study demonstrates that CALB2 expression is associated with tumor progression and unfavorable prognosis in colorectal adenocarcinoma. Clinical analyses, together with functional experiments, indicate that altered CALB2 expression is linked to enhanced proliferative and migratory behaviors, as well as increased tumor growth in vivo. In addition, CALB2 expression was accompanied by changes in EMT-related markers, p53 signaling activity, and amino acid metabolic profiles. Although the underlying mechanisms require further investigation, these findings suggest that CALB2 may have potential relevance as a progression-associated biomarker in colorectal adenocarcinoma.

## Data Availability

Additional data supporting the findings of this study are available from the corresponding author upon reasonable request.

## Supplementary Materials

The following supporting information can be downloaded at https://www.mdpi.com/article/10.3390/genes17050510/s1. Figure S1: Time-course analysis of CALB2 knockdown efficiency in HCT116 cells; Figure S2: The second independent CALB2 siRNA (si2-CALB2) suppresses cell proliferation in HCT116 and HT29 cells (CCK-8 assays); Figure S3: The second independent CALB2 siRNA (si2-CALB2) inhibits cell migration in HCT116 and HT29 cells (wound-healing and Transwell assays); Table S1: Correlation between CALB2 expression and clinicopathological parameters in COAD patients (n = 30); Full uncropped Western blot images for Figure 2, Figure 5 and Figure 6.

## Abbreviations

The following abbreviations are used in this manuscript:

ATCC	American Type Culture Collection
CALB2	Calbindin 2
CCK-8	Cell Counting Kit-8
COAD	Colorectal Adenocarcinoma
EMT	Epithelial–Mesenchymal Transition
KEGG	Kyoto Encyclopedia of Genes and Genomes
LC–MS	Liquid Chromatography–Tandem Mass Spectrometry
qRT-PCR	Quantitative Reverse Transcription PCR
TCGA	The Cancer Genome Atlas
WB	Western Blot
