# Supplementary material for: CALB2 Expression Is Associated with Tumor Progression and Prognosis in Colorectal Adenocarcinoma
Source: Genes (Basel). 2026 Apr 25;17(5):510. doi: 10.3390/genes17050510 (PMC13206491; doi:10.3390/genes17050510)
Supplement: Supplementary file 1 [file genes-17-00510-s001.zip › Supplementary figures and legends.pdf]

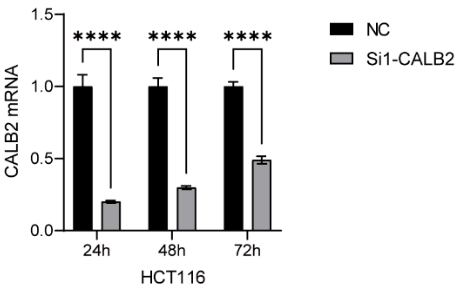

Supplementary Figure S1. Time-course analysis of CALB2 knockdown efficiency. Relative CALB2 mRNA expression was evaluated by qRT-PCR in HCT116 cells at 24, 48, and 72 hours following transfection with CALB2 siRNA (Si1-CALB2) or a negative control (NC). The data demonstrate that significant mRNA suppression is achieved at 24 hours and stably maintained through 72 hours. (n = 3, **** $P < 0.0001$ vs. NC group).

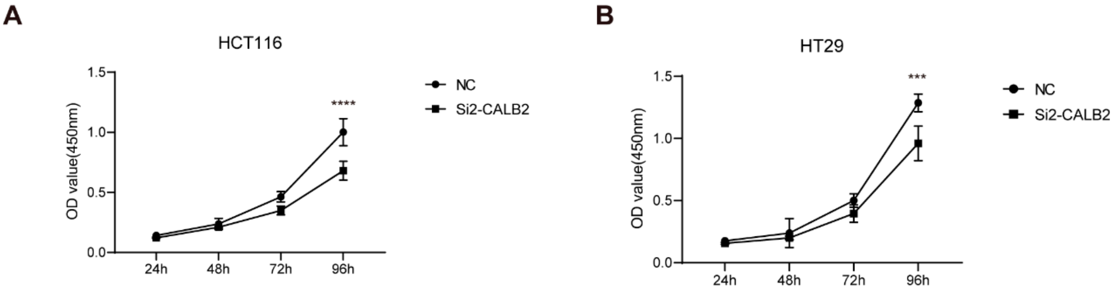

Supplementary Figure S2. The second independent CALB2 siRNA produces consistent inhibitory effects on cell proliferation. (A, B) Cell proliferation of HCT116 (A) and HT29 (B) cells following transfection with a negative control (NC) or the second independent CALB2 siRNA (Si2-CALB2) was assessed by CCK-8 assays at 24, 48, 72, and 96 hours. (n = 3, *** $P < 0.001$, **** $P < 0.0001$ vs. NC group).

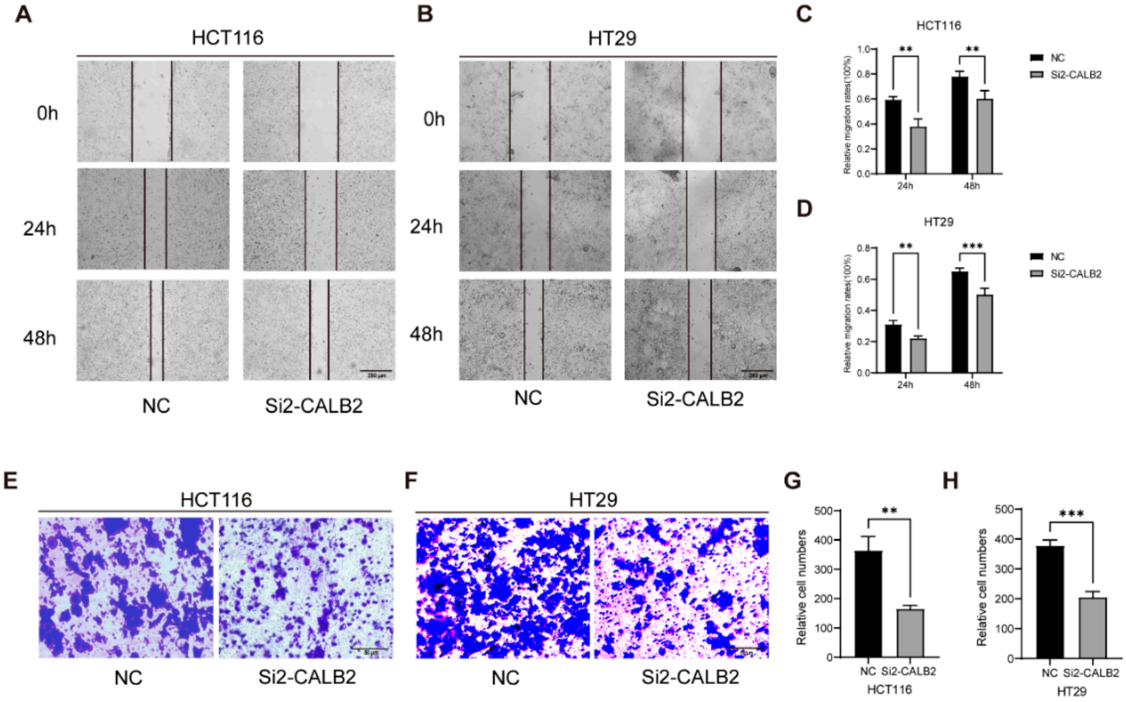

Supplementary Figure S3. The second independent CALB2 siRNA significantly inhibits the

migration of colorectal adenocarcinoma cells. (A, B) Representative images of wound healing assays in HCT116 (A) and HT29 (B) cells transfected with a negative control (NC) or the second independent CALB2 siRNA (Si2-CALB2). Images were captured at 0, 24, and 48 hours post-scratching. (C, D) Quantification of relative migration rates (wound closure) in HCT116 (C) and HT29 (D) cells at 24 and 48 hours. (E, F) Representative images of Transwell migration assays in HCT116 (E) and HT29 (F) cells following NC or Si2-CALB2 transfection. (G, H) Quantification of the relative number of migrated cells in the Transwell assays for HCT116 (G) and HT29 (H). (n = 3, ** $P < 0.01$, *** $P < 0.001$ vs. NC group).
