# Supplementary material for: CALB2 Expression Is Associated with Tumor Progression and Prognosis in Colorectal Adenocarcinoma
Source: Genes (Basel). 2026 Apr 25;17(5):510. doi: 10.3390/genes17050510 (PMC13206491; doi:10.3390/genes17050510)
Supplement: Supplementary file 1 [file genes-17-00510-s001.zip › Supplementary Table 1.pdf]

Supplementary Table S1: Correlation between CALB2 expression and clinicopathological parameters in COAD patients (n = 30).

Characteristic	Category	n	CALB2-positive cells (%, Mean ± SD)	*P* value
Age (years)	< 60	22	68.32 ± 19.85	0.482
	≥ 60	8	74.56 ± 22.14	
Gender	Male	20	70.18 ± 20.42	0.865
	Female	10	69.59 ± 21.36	
Tumor Size (cm)	< 4.0 cm	9	66.85 ± 22.45	0.526
	≥ 4.0 cm	21	71.32 ± 19.68	

P values were calculated using the Mann-Whitney U test.
