# Supplementary figures and images for: CALB2 Expression Is Associated with Tumor Progression and Prognosis in Colorectal Adenocarcinoma
Source: Genes (Basel). 2026 Apr 25;17(5):510. doi: 10.3390/genes17050510 (PMC13206491; doi:10.3390/genes17050510)

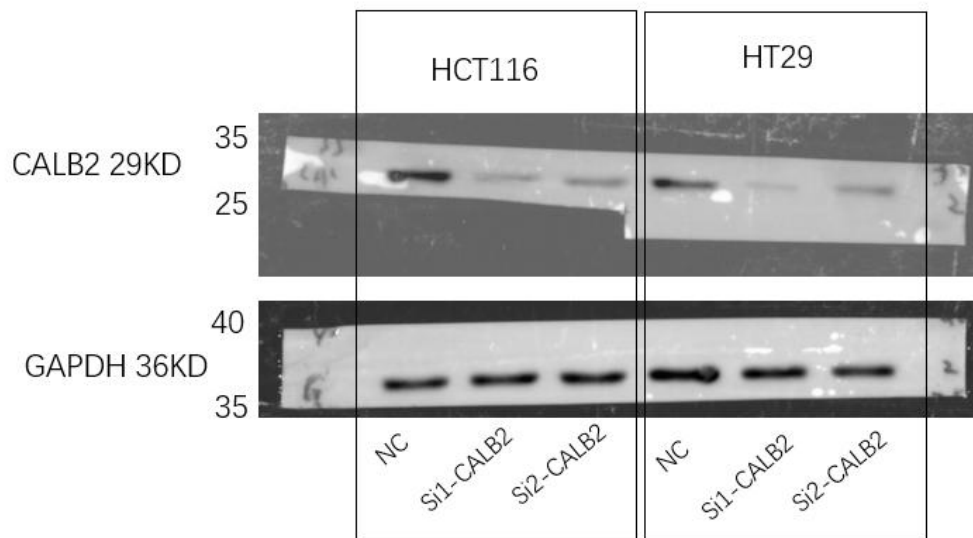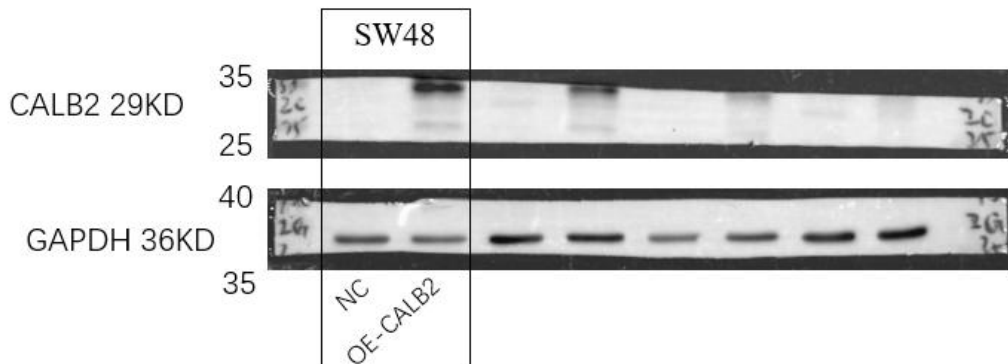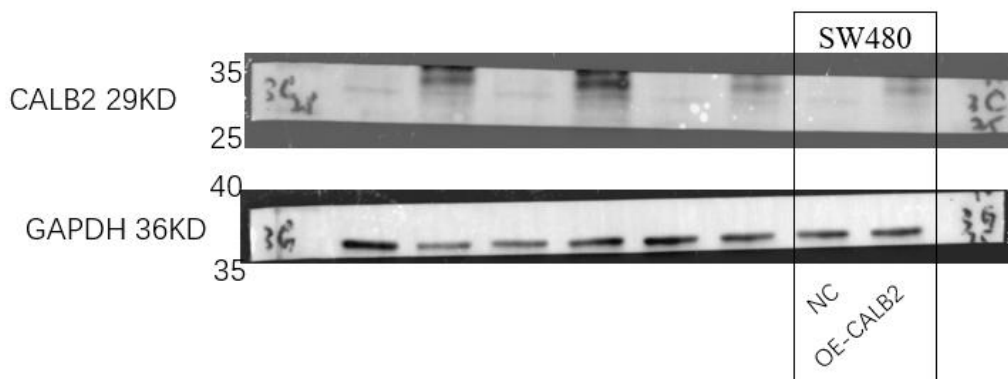

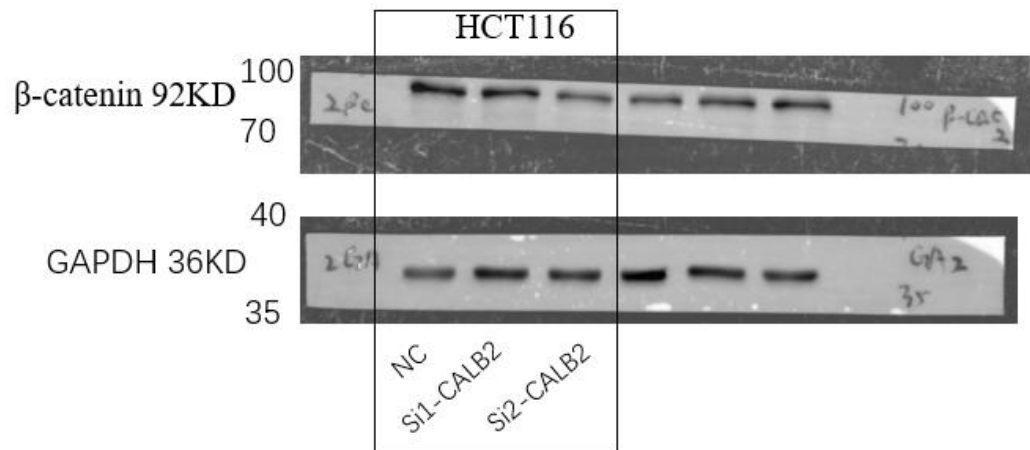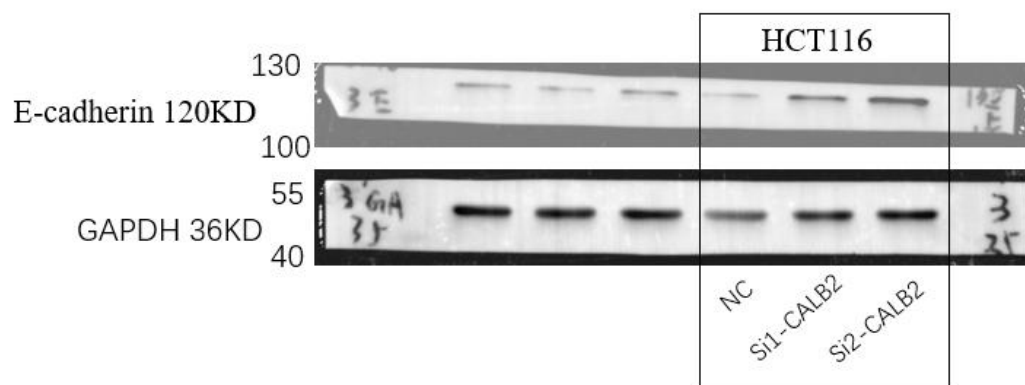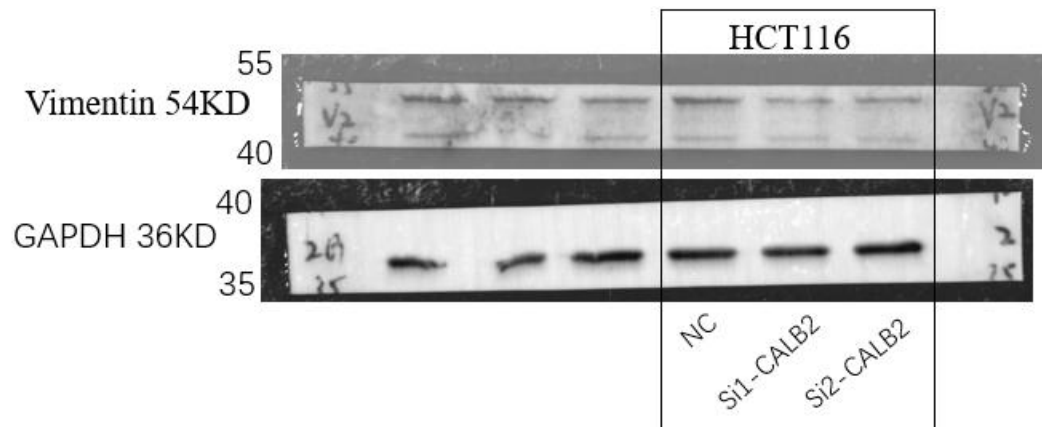

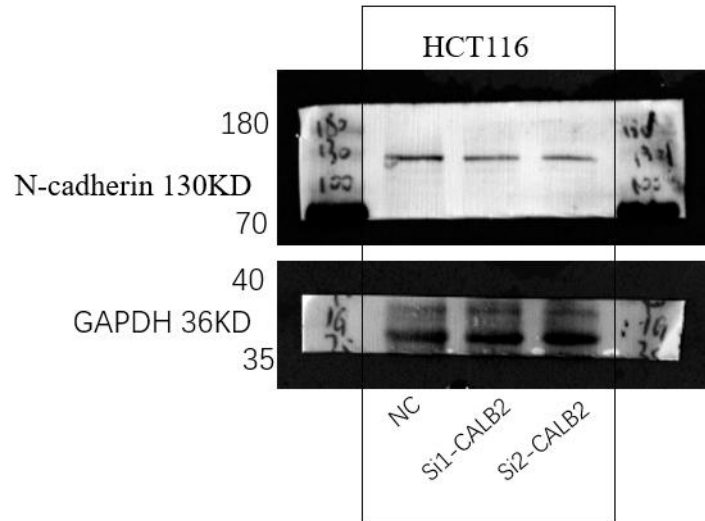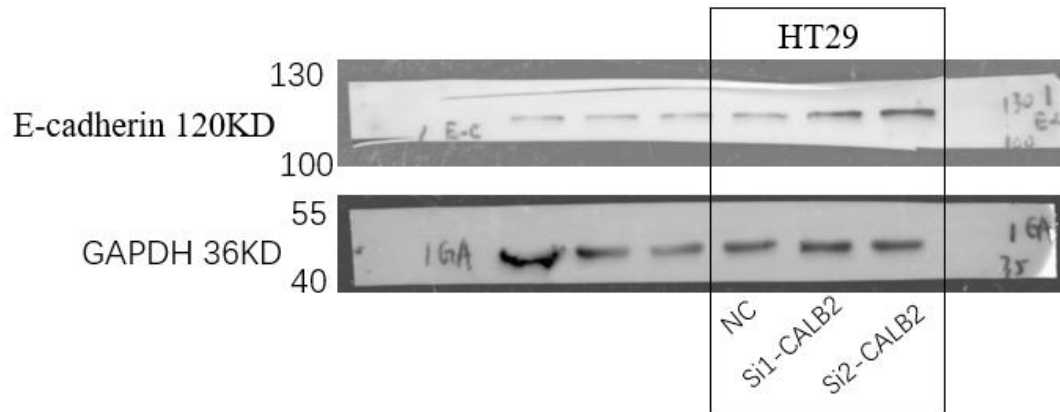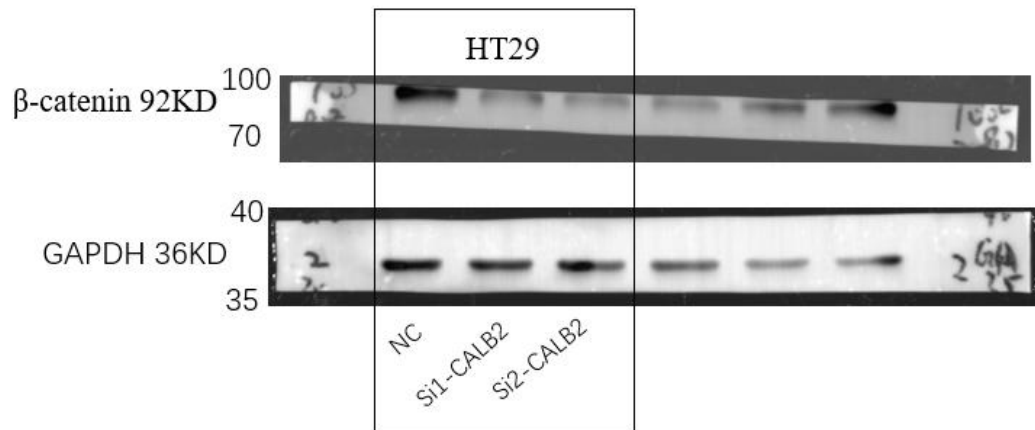

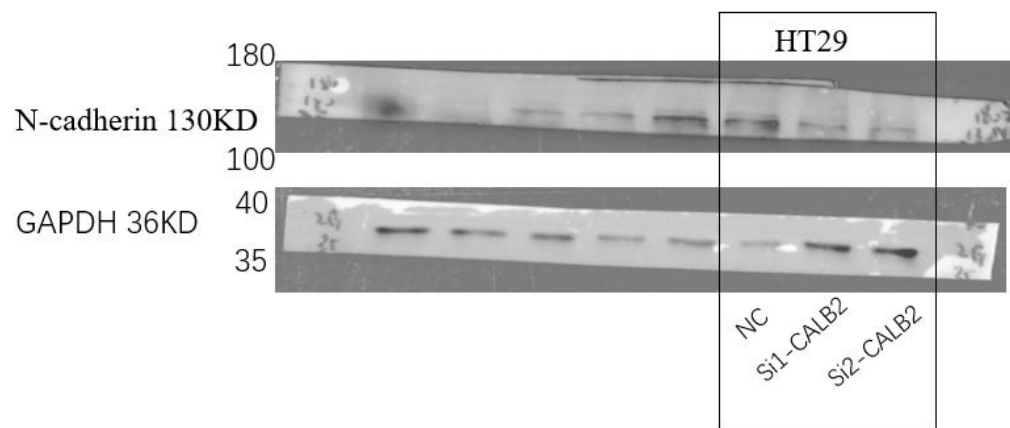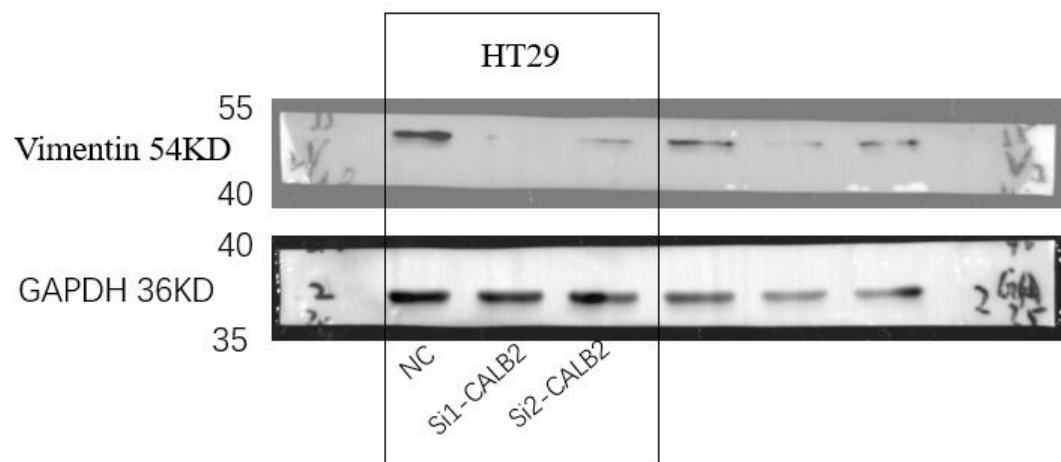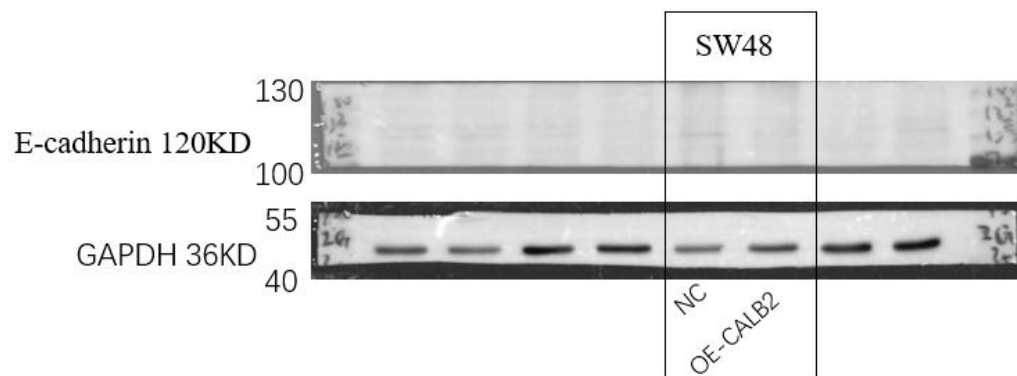

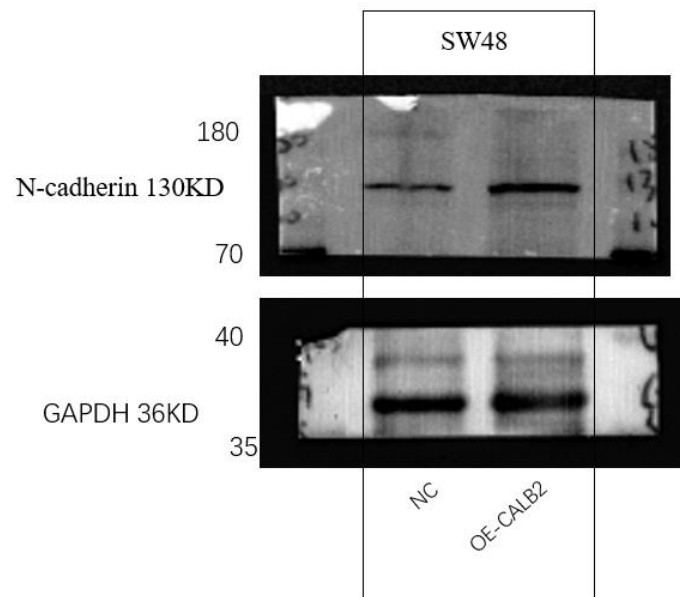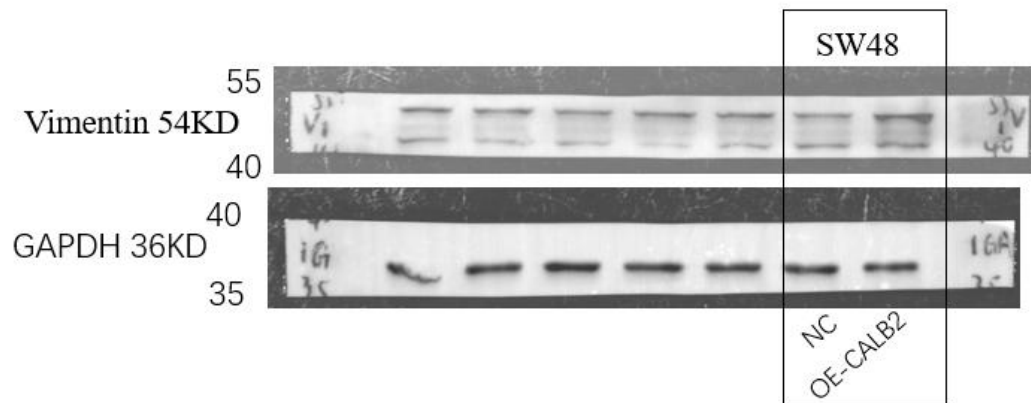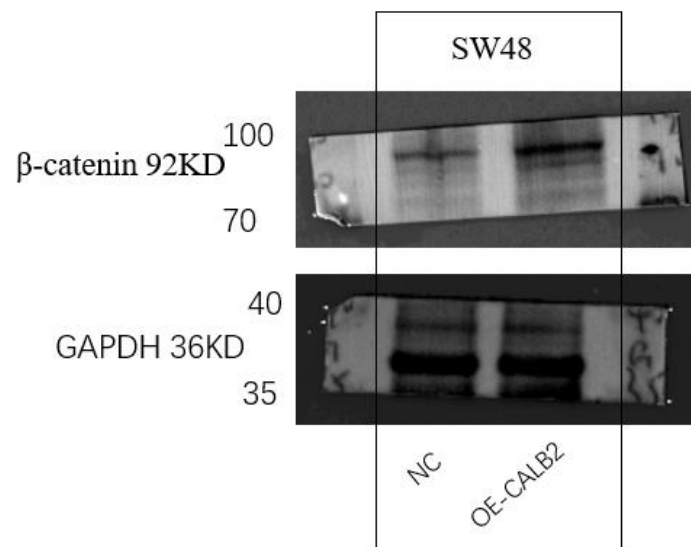

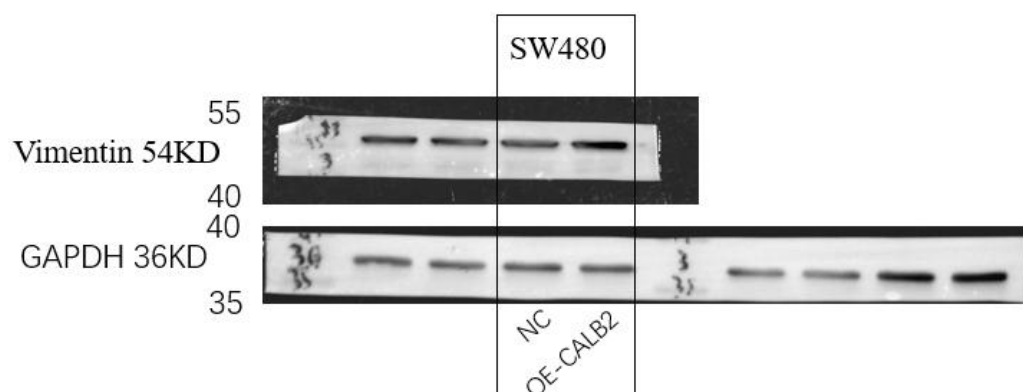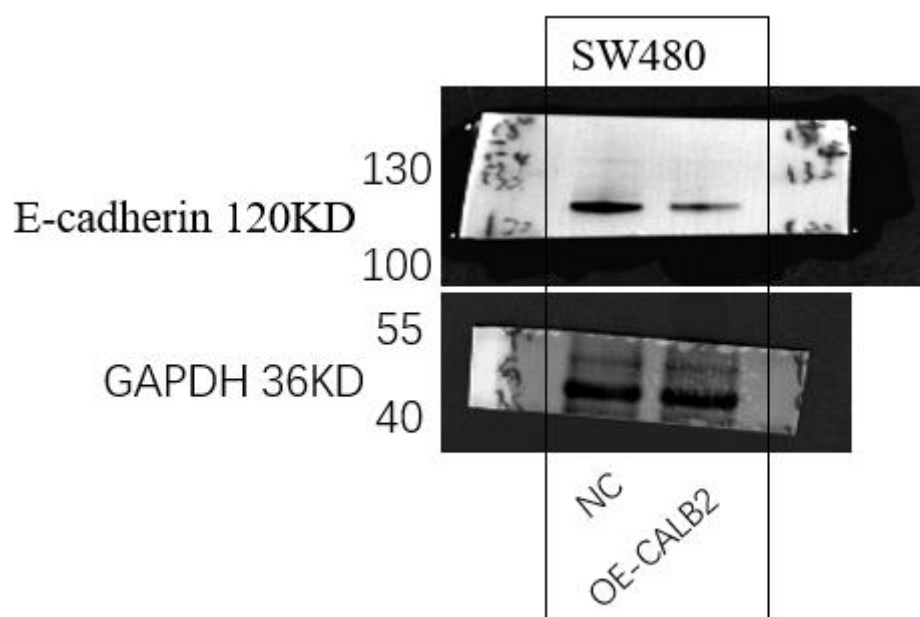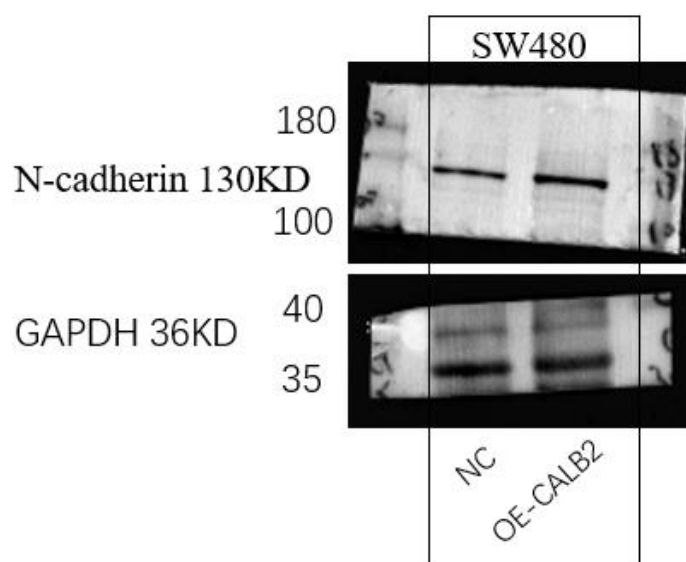

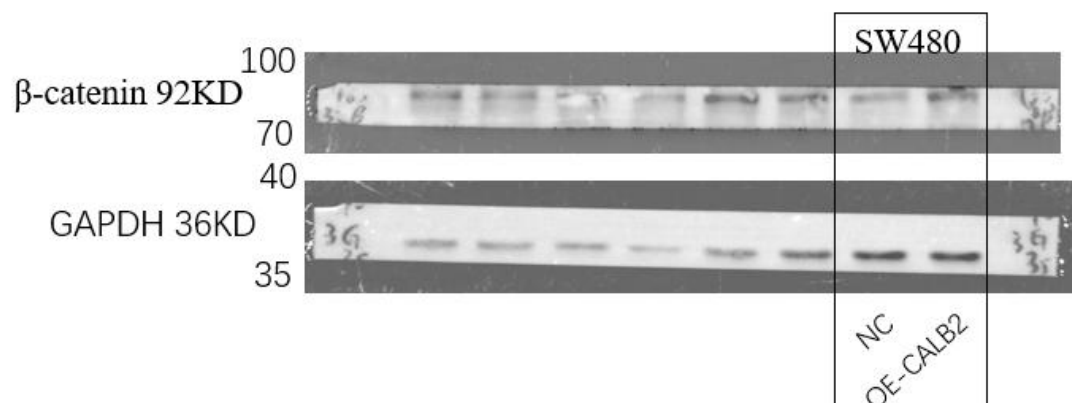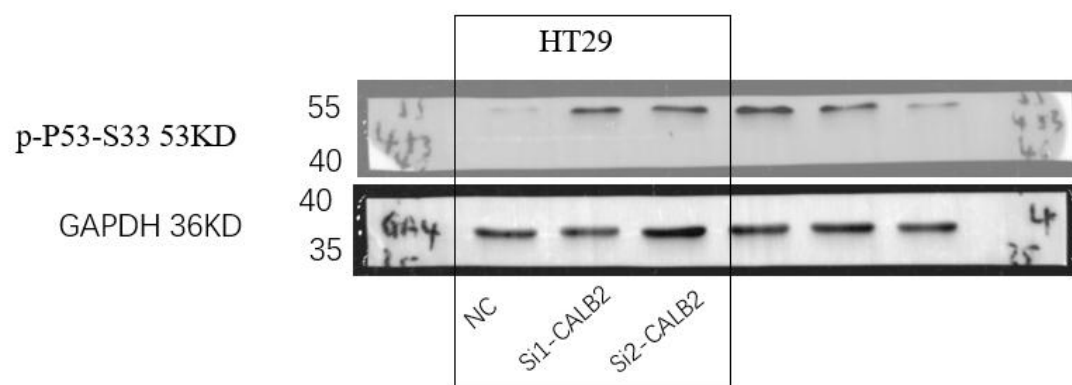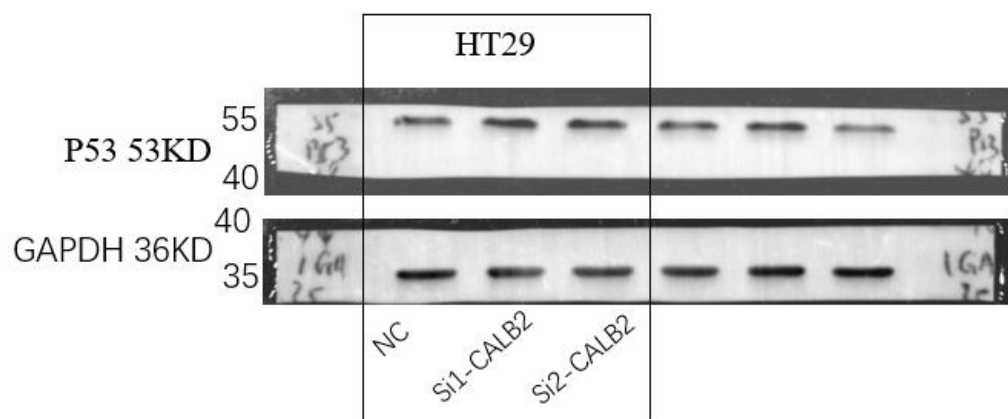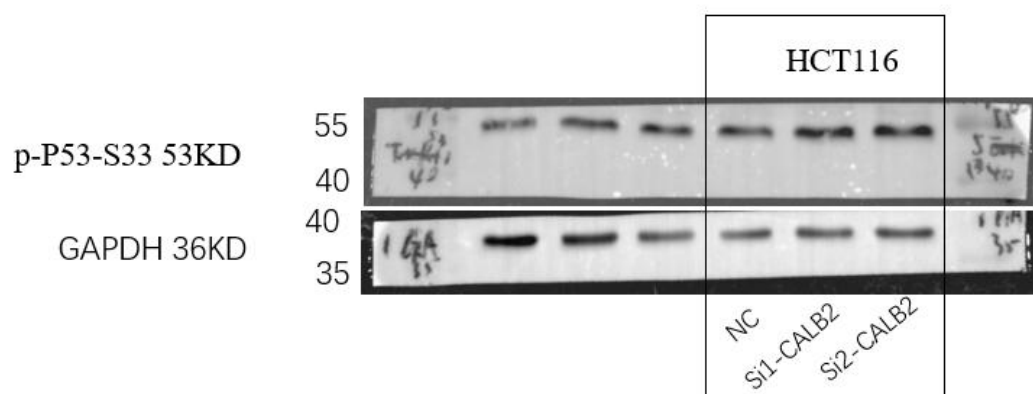

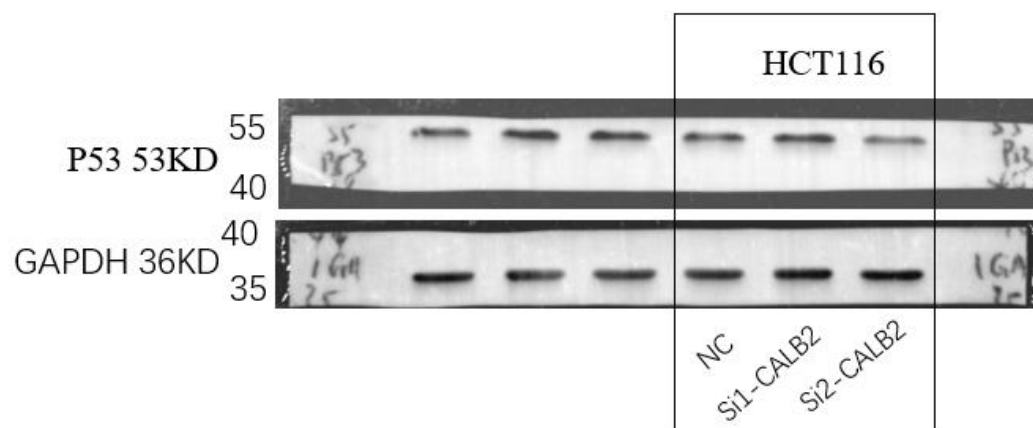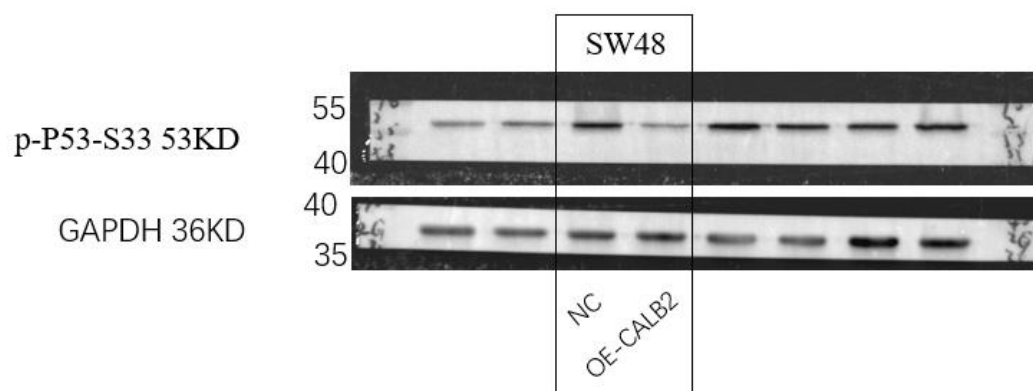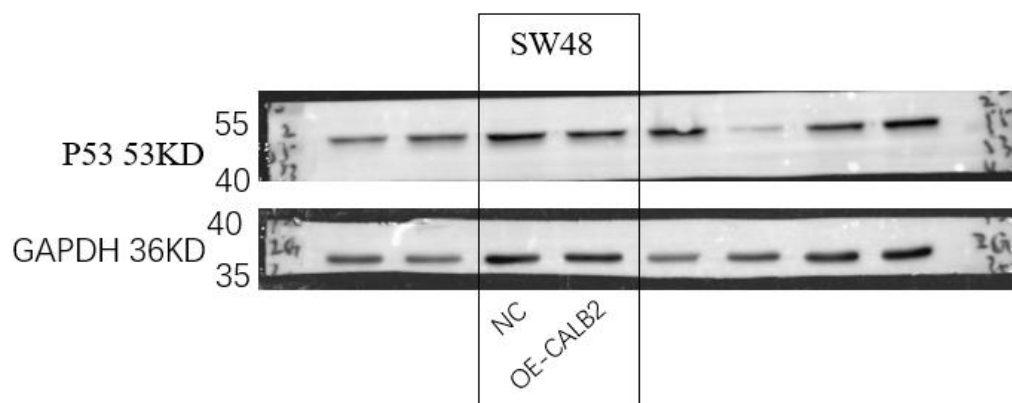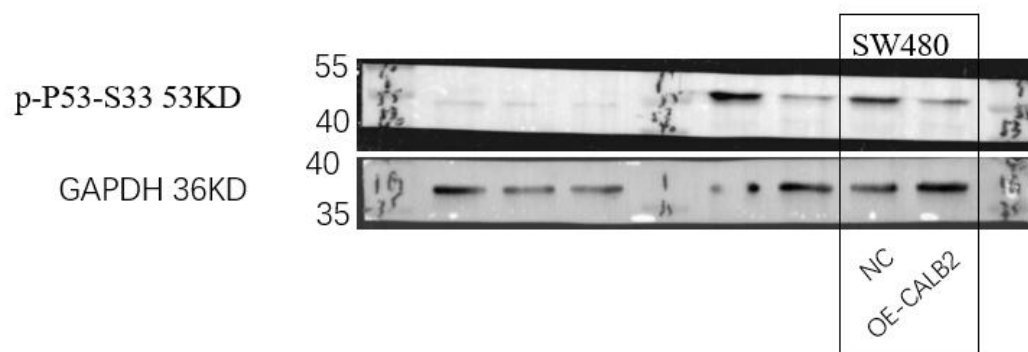

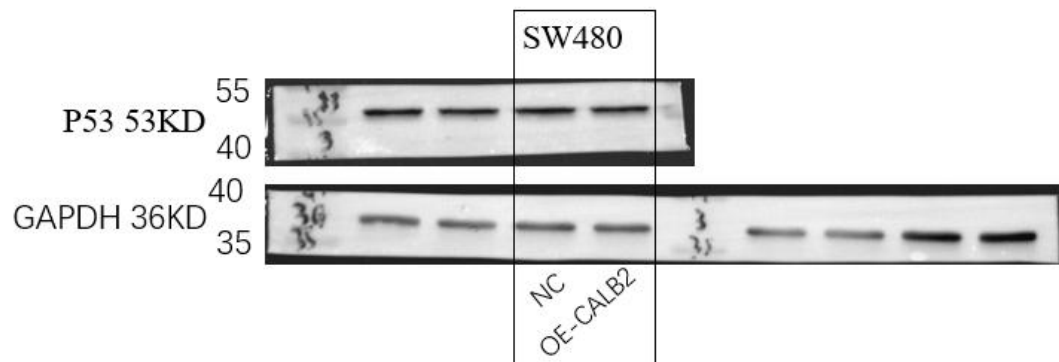

Supplement: Supplementary file 1 [file genes-17-00510-s001.zip › full uncropped Gels and Blots image(s).pdf]
